# Phase I Clinical Trial of Systemically Administered *TUSC2(FUS1)-*Nanoparticles Mediating Functional Gene Transfer in Humans

**DOI:** 10.1371/journal.pone.0034833

**Published:** 2012-04-25

**Authors:** Charles Lu, David J. Stewart, J. Jack Lee, Lin Ji, Rajagopal Ramesh, Gitanjali Jayachandran, Maria I. Nunez, Ignacio I. Wistuba, Jeremy J. Erasmus, Marshall E. Hicks, Elizabeth A. Grimm, James M. Reuben, Veerabhadran Baladandayuthapani, Nancy S. Templeton, John D. McMannis, Jack A. Roth

**Affiliations:** 1 The University of Texas MD Anderson Cancer Center, Houston, Texas, United States of America; 2 University of Oklahoma Health Sciences Center and Stephenson Cancer Center, Oklahoma City, Oklahoma, United States of America; 3 Gradalis Inc, Carrollton, Texas, United States of America; National Cancer Center, Japan

## Abstract

**Background:**

Tumor suppressor gene *TUSC2/FUS1 (TUSC2)* is frequently inactivated early in lung cancer development. *TUSC2* mediates apoptosis in cancer cells but not normal cells by upregulation of the intrinsic apoptotic pathway. No drug strategies currently exist targeting loss-of–function genetic abnormalities. We report the first in-human systemic gene therapy clinical trial of tumor suppressor gene *TUSC2*.

**Methods:**

Patients with recurrent and/or metastatic lung cancer previously treated with platinum-based chemotherapy were treated with escalating doses of intravenous *N*-[1-(2,3-dioleoyloxy)propyl]-*N*,*N*,*N*-trimethylammonium chloride (DOTAP):cholesterol nanoparticles encapsulating a *TUSC2* expression plasmid (DOTAP:chol-*TUSC2*) every 3 weeks.

**Results:**

Thirty-one patients were treated at 6 dose levels (range 0.01 to 0.09 milligrams per kilogram). The MTD was determined to be 0.06 mg/kg. Five patients achieved stable disease (2.6–10.8 months, including 2 minor responses). One patient had a metabolic response on positron emission tomography (PET) imaging. RT-PCR analysis detected *TUSC2* plasmid expression in 7 of 8 post-treatment tumor specimens but not in pretreatment specimens and peripheral blood lymphocyte controls. Proximity ligation assay, performed on paired biopsies from 3 patients, demonstrated low background TUSC2 protein staining in pretreatment tissues compared with intense (10–25 fold increase) TUSC2 protein staining in post-treatment tissues. RT-PCR gene expression profiling analysis of apoptotic pathway genes in two patients with high post-treatment levels of TUSC2 mRNA and protein showed significant post-treatment changes in the intrinsic apoptotic pathway. Twenty-nine genes of the 82 tested in the apoptosis array were identified by Igenuity Pathway Analysis to be significantly altered post-treatment in both patients (Pearson correlation coefficient 0.519; p<0.01).

**Conclusions:**

DOTAP:chol-*TUSC2* can be safely administered intravenously in lung cancer patients and results in uptake of the gene by human primary and metastatic tumors, transgene and gene product expression, specific alterations in *TUSC2*-regulated pathways, and anti-tumor effects (to our knowledge for the first time for systemic DOTAP:cholesterol nanoparticle gene therapy).

**Trial Registration:**

ClinicalTrials.gov NCT00059605

## Introduction

The focus of cancer therapy has shifted from the tissue to the genetic level. [1] Mutations in two major classes of genes, oncogenes and tumor suppressor genes (TSGs), play central roles in the oncogenic process. TSGs appear to require homozygous deletion or mutation for inactivation, [2] and restoration of TSG expression is feasible in human tumors. [3] Intratumoral injection of retroviral or adenoviral vectors expressing the wildtype TSG p53 have been performed in patients with locally advanced non-small cell lung cancer (NSCLC) and head and neck cancer. [4], [5], [6] These studies showed that viral vectors expressing the TSG p53 can be safely injected into tumors repetitively and can mediate tumor regression. However, because of the systemic immune response, current viral vectors are limited to intratumoral administration or single intravenous doses, which may not be optimal treatment for tumor metastases, the primary cause of cancer-related death. Thus development of vectors for intravenous, systemic TSG replacement would represent a significant advance. Homozygous deletions in the 3p21.3 region in lung cancer cell lines and primary lung tumors have led to the identification of multiple genes with tumor suppressor activity. [7] Among these genes, *TUSC2*, consistently demonstrates the highest level of tumor suppressor activity. [8] Here we report the first-in-human, dose-escalation clinical trial of intravenous DOTAP:cholesterol(DC)-*TUSC2* in patients with incurable, recurrent and/or metastatic lung cancer previously treated with platinum-based chemotherapy. The primary objectives of the clinical trial were to assess the toxicity of DOTAP:chol-*TUSC2* administered intravenously and to determine the maximal tolerated dose and recommended phase II dose of DOTAP:chol-*TUSC2* administered intravenously. Secondary objectives were to assess the expression of *TUSC2* following intravenous delivery of DOTAP:chol-*TUSC2* in tumor biopsies and assess any anti-cancer activity for DOTAP:chol-*TUSC2*. This is the first, to our knowledge, systemic gene therapy clinical trial that uses an intravenous nanoparticle-delivery system for delivering a tumor suppressor gene expression plasmid (Phase I Study of IV DOTAP: Cholesterol-Fus1 in Non-Small-Cell Lung Cancer.

## Methods

Please see Information S1 for detailed methods.

### Trial Protocol

The protocol for this trial is available as Protocol S1.

### DC nanoparticle preparation

DOTAP GMP grade was purchased from Avanti Polar Lipids, Inc. (Alabaster, AL) and cholesterol GMP grade was purchased from Sigma-Aldrich (St. Louis, MO). A ratio of 20 mM DOTAP:18 mM cholesterol was used for preparation of the nanoparticles. The reagents were mixed and the dry lipids dissolved in purified GMP grade chloroform. A Buchi rotary evaporator was used to form a dry lipid film. Further drying was performed under a vacuum in a Labconco Freeze dry system. The film was resuspended in sterile 5% dextrose in water. After sonication the following day under aseptic conditions the lipids are sequentially extruded through a series of sterile Whatman filters from 1 um to 0.1 um in pore size.

### Plasmid production

The pLJ143-KGB2-*TUSC2* plasmid vector was produced under GMP conditions at the Baylor College of Medicine Center for Cell and Gene Therapy (Houston, TX) and the Beckman Research Institute of the City of Hope (Duarte, CA). The toxicity profiles did not differ for the two production lots (Table S1 – Dose-escalation scheme). Both lots had similar quality control specifications (Table S2 – Plasmid quality control specifications). The plasmid structure, restriction enzyme map, and sequence are shown in Figures S1 (Structure and restriction enzyme map of pLJ143/KGB2/FUS1 plasmid vector) and Figure S2 (Positions and direction of DNA sequencing primers for pLJ143/KGB2/FUS1 vector) and Table S3(DNA sequence of pLJ143/KGB2/FUS1).

### DC-*TUSC2* nanoparticle preparation

The diluted plasmid DNA and diluted nanoparticle stock were mixed in equal volumes to a final concentration of 4 mM DOTAP and 0.5 mg/ml of DNA. Prior to treatment the assigned dose was diluted in 100 ml 5% dextrose in water (D5W). A negative gram stain was required prior to treatment.

### Study Design

Eligible patients were required to have histologically documented NSCLC or SCLC not curable by standard therapies and previously treated with platinum-based chemotherapy. Cycles consisted of a single intravenous infusion every 21 days. The presence of viable cancer cells in the biopsied lesion was confirmed in all cases by histopathological examination. Mandatory biopsies for gene expression analysis were explicitly precluded by regulatory committees at the local and federal level. Tumor response assessed by computed tomography (CT) scans was determined in accordance with standard World Health Organization (WHO) criteria. [9] This study was approved by the University of Texas MD Anderson Institutional Review Board, the NIH Recombinant DNA Advisory Committee, and the FDA. All patients provided written informed consent prior to entry into the study.

### Expression of *TUSC2* plasmid in human tumors

Tissue was obtained by 20 gauge needle core biopsy using CT guidance. All specimens were blinded for patient identity, clinical information and specimen timing (pre- vs post-treatment) during all studies. Ectopic expression of the *TUSC2* gene in patient biopsy samples was analyzed using a TaqMan based quantitative real time reverse transcriptase-polymerase chain reaction (RT-PCR) (Applied Biosystems, Foster City, CA).

### *In situ* Proximity Ligation Assay (PLA) for TUSC2 protein in tumor biopsies

The rabbit anti-TUSC2 polyclonal antibody used for immunohistochemical staining was raised against a synthetic oligopeptide derived from the TUSC2 NH_2_-terminal amino acid sequence (NH_2_-GASGSKARGLWPFAAC). The specificity of this antibody for TUSC2 was previously characterized. [10], [11] Duolink kits from Olink Biosciences (Uppsala, Sweden) were used to analyze changes in TUSC2 protein expression in pre- and post-treatment tumor biopsies.

### Statistical Analysis

The initial starting dose (0.02 mg/kg) was selected based on toxicology studies in non-human primates. This dose was one tenth the dose which resulted in no deaths in non-human primates. After the sixth patient was enrolled, the starting dose was amended to 0.01 mg/kg. Dose escalation was based on a continuous reassessment method (CRM) which allows the MTD to be periodically re-estimated (see Protocol S1 for complete description) [12] The MTD was defined as the highest dose level in which no more than 10% of patients develop dose-limiting toxicity (DLT), defined as grade 3 non-hematologic or hematologic toxicity during cycle 1 judged by the investigator to be related to DOTAP:chol-TUSC2. Patients entered at a given dose level were not eligible for dose escalation or dose reduction. A cohort of 3 patients was treated at each dose level. After treating 3 patients at a given dose level, the information of whether the patients developed DLT was used to compute the posterior probability of toxicity. Only toxicity during cycle 1 was used to determine the next dose level. If no DOTAP:chol-TUSC2-related toxicities were observed in any prior patient, the subsequent dose level was increased by 100%. If only grade 1 or 2 toxicities were observed, the subsequent dose level was increased by 50%. If any DLT was observed, the CRM could lead to either escalation or reduction of dose levels. If DLT occurred and the CRM resulted in a dose escalation, the subsequent dose level was increased by 25%. The computer program crm.exe was used (http://odin.mdacc.tmc.edu/anonftp/). Toxicity was graded according to the National Cancer Institute Common Toxicity Criteria, version 2.0. Tumor status was assessed at baseline and after every two cycles of therapy with computed tomography (CT) scans and/or positron emission tomography (PET)/CT scans. Expression of major genes in apoptosis signaling pathway**s** in tumor fine needle biopsies from human lung cancer patients before and after systemic treatment with DOTAP:chol-TUSC2 nanoparticles were quantified using a qRT Profiler Apoptosis PCR Array with RT Nano PreAmp-mediated cDNA synthesis (SA Biosciences, Frederick, MD). The quantitative apoptotic gene expression data were analyzed through the use of Ingenuity Pathway Analysis (Ingenuity Systems, Inc. Redwood City CA (www.ingenuity.com) (IPA)). For the network and canonical pathway analysis, the quantitative PCR data set containing gene identifiers and corresponding expression fold change values was uploaded into the application. Each identifier was mapped to its corresponding object in Ingenuity's Knowledge Base (IKB). An expression fold change (posttreatment/pretreatment) cutoff of 3 was set to identify molecules whose expression was significantly differentially regulated. These molecules, called Network Eligible molecules, were overlaid onto a global molecular network developed from information contained in IKB. Networks of Network Eligible Molecules were then algorithmically generated based on their direct or indirect connectivity. The Network molecules associated with biological functions in IKB were considered for analysis. Right-tailed Fisher's exact test was used to calculate a p-value determining the probability that each biological function assigned to a given network is due to chance alone. Molecules from the data set that met the above gene expression fold changes cutoff were also considered for the canonical pathway analysis. The significance of the association between the data set and the canonical pathway was measured by a ratio of the total number of molecules from the data set that map to the pathway to the total number of molecules that map to the canonical pathway in IKB. A Fisher's exact test was used to calculate a p-value determining the probability that the association between the genes in the dataset and the canonical pathway is explained by chance alone. A binomial sampling model using an exact test of proportions, was used to test the null hypothesis that coordinated gene expression for both patients occurred by chance using the R statistical package (http://cran.r-project.org/). Comparisons in the PLA were are by two-tailed unpaired Student's t-test assuming equal variances as determined by F test using GraphPad Prism software (GraphPad Software, Inc., La Jolla, CA). Median survival was determined by the Kaplan Meier method using SPSS statistical software version 15 (IBM, Armonk, NY).

## Results

**Figure 1:**
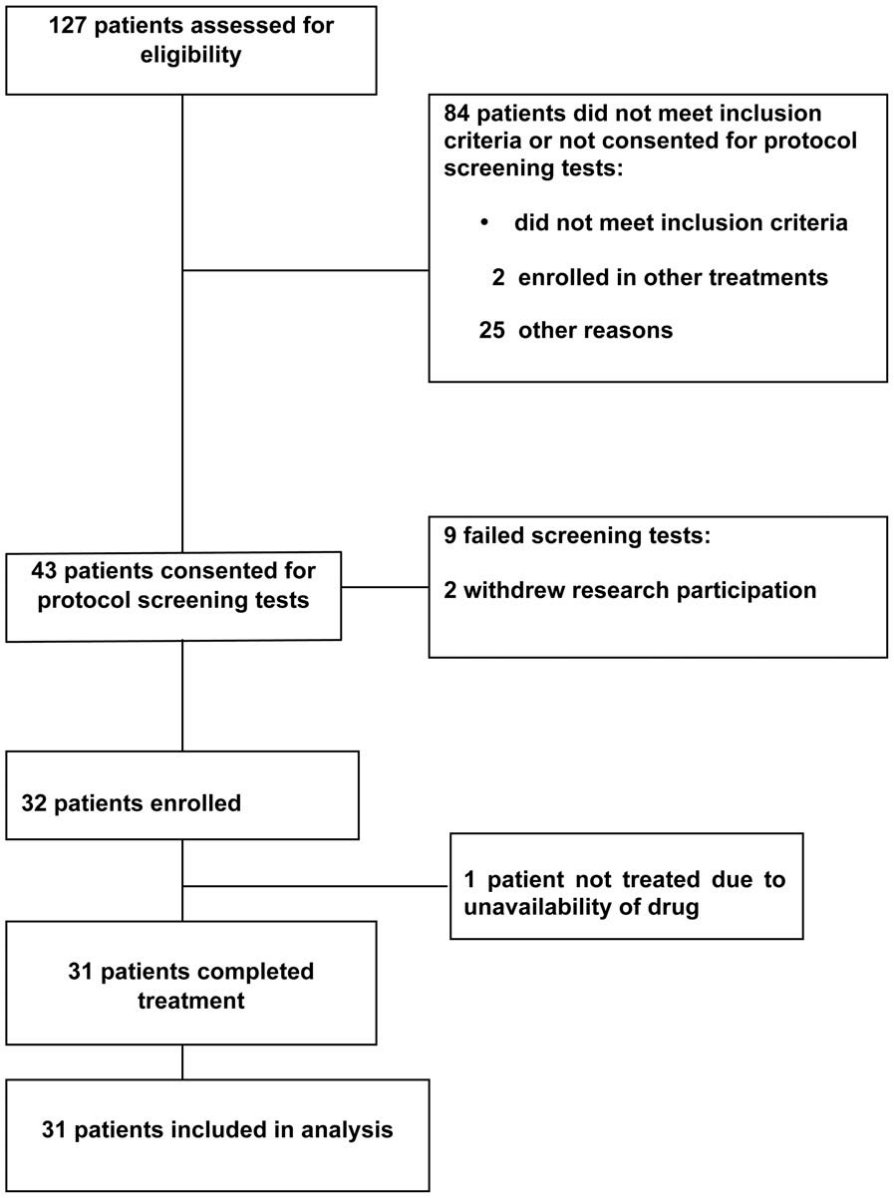
Consort style flowchart of participants through the study.

One hundred and twenty seven patients were assessed for eligibility, 31 patients were enrolled between May 5, 2003 and January 6, 2010 and all patients in the study were treated at a single institution (Figure 1). Patient characteristics are described in Table 1. The IRB did not permit mandatory tumor biopsies; however, eight patients consented to pre and post-treatment tumor biopsies.

**Table 1:** Baseline Characteristics of Patients.

Characteristic	No. of Patients (%) (n = 31)
Median age, years (range)	60 (43–76)
Sex	
Male	16 (52%)
Female	15 (48%)
ECOG performance status	
0	4 (13%)
1	27 (87%)
Histology	
Adenocarcinoma	11 (35%)
Bronchoalveolar carcinoma	1 (3%)
Squamous cell carcinoma	3 (10%)
Non-small cell carcinoma, NOS	11 (36%)
Small cell carcinoma	5 (16%)
Prior therapy	
Chemotherapy	31 (100%)
Radiotherapy	14 (45%)
Surgery	11 (35%)
Prior chemotherapy regimens	
1	9 (29%)
2	9 (29%)
>2	13 (42%)
Number of doses received	
1	8 (26%)
2	19 (61%)
≥3	4 (13%)

Abbreviations: ECOG, Eastern Cooperative Oncology Group; NOS, not otherwise specified.

### Toxicity and Maximum Tolerated Dose (MTD) Determination

A total of 74 cycles of DC-*TUSC2* were administered, with a median of 2 cycles (range, 1 to 12 cycles) per patient. Patients were treated at 6 dose levels ranging from 0.01 to 0.09 mg/kg. The dose escalation scheme, including number of patients, number of cycles, and toxicities judged to be related to DC-*TUSC2* are listed in Table 2. Twenty-three patients received two or more doses. The first patient in cohort 1 (receiving 0.02 mg/kg) developed grade 2 fever within 3 hours of the DC-*TUSC2* infusion. The subsequent patients in cohorts 1 and 2 were given dexamethasone and diphenhydramine prior to receiving DC-*TUSC2*, and no grade 1 or higher toxicites were observed. However, after discussions with the FDA, it was mandated that the next patient cohort receive DC-*TUSC2* at a lower dose level of 0.01 mg/kg without dexamethasone or diphenhydramine premedication. All three patients developed grade 2 or 3 fever and one patient developed grade 3 hypotension. The FDA then allowed the protocol to be amended to require dexamethasone and diphenhydramine premedications beginning with the next cohort (patient 10), starting at a dose level of 0.01 mg/kg. Due to this amendment, it was decided not to use the toxicity data from the first nine patients for MTD determination, and a subgroup of 21 patients enrolled between September 28, 2006 and October 29, 2009 were used to determine the final MTD. The only subsequent dose limiting toxicities (DLT) observed were grade 3 reversible hypophosphatemia in two patients with one at 0.06 mg/kg and another at 0.09 mg/kg. In both cases the patients had either grade 1 or 2 fevers and the hypophosphatemia was an incidental laboratory finding. The MTD was determined to be 0.06 mg/kg. As listed in Table 2, grade 2 toxicities included myalgias, hypophosphatemia, fever, nausea, and transaminase elevation. Antibodies to single and double stranded DNA were not detected 14 months after completion of 12 cycles of therapy in patient 26. At the time of entry into the study all patients had tumor progression despite multiple systemic therapies and had growing tumor(s).

**Table 2:** Dose-Escalation Scheme, Toxicities, and Tumor Response.

Cohort No.	Dose level (mg/kg)	No. of Patients	No. of cycles	No. patients with DLT	Grade 2 toxicity (No. patients)	Tumor Response (No. of patients)
1	0.02	3	9	0	Fever (1)	SD (1)
2	0.03	3	6	0	0	SD (1)
3^1^	0.01	3	4	2; G3 fever (n = 2), G3 hypotension (n = 1)	Fever (1)	
4*	0.01	3	9	0	0	SD (1)^2^
5*	0.02	3	6	0	0	
6*	0.04	3	6	0	0	
7*	0.06	3	6	0	ALT (1), neuropathy (n = 1)	SD(1)^3^
8*	0.09	3	5	1; G3 hypophosphatemia	Fever (1)	
9*	0.06	3	16	0	Hypophosphatemia (1), nausea (1), myalgia (1)	SD(1)^4^
10*	0.06	3	5	1; G3 hypophosphatemia	Fever (1), myalgia (1), hypophosphatemia (1),	
11	0.06	1	2	0	0	

Abbreviations: G3, grade 3; ALT, alanine aminotransferase elevation; SD, stable disease.

1 This cohort did not receive dexamethasone or diphenhydramine premedications.

2 reduction in primary tumor size of 14%.

3 reduction in primary tumor size of 26%.

4 metabolic response (Figure 3).

* Cohorts used to determine maximum tolerated dose (MTD).

### Response and survival

**Figure 2:**
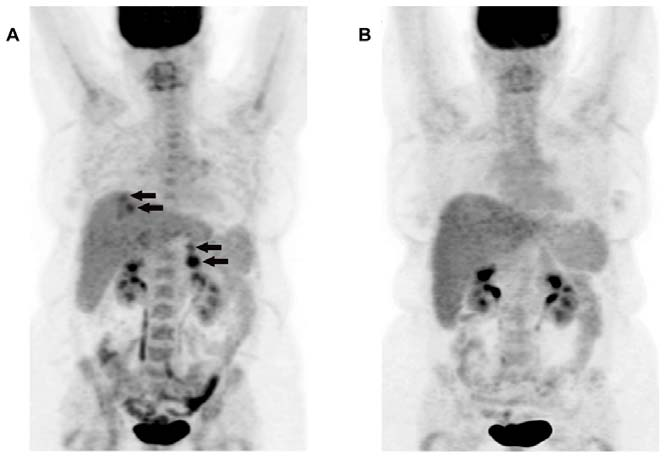
DC-*TUSC2* metabolic tumor response in a metastatic lung cancer patient. The patient is a 54 year old female with a large cell neuroendocrine carcinoma. She had received six prior chemotherapy regimens. Prior to entry in the protocol, two hepatic metastases were progressing on gemcitabine. The patient also had a metastasis in the head of the pancreas and a peripancreatic lymph node (arrows). A. Pretreatment PET scan. The dose of Fluorodeoxyglucose(18F) was 8.8mCi B. Post-treatment PET scan performed 20 days following the fourth dose of DC-*TUSC2*. The dose of Fluorodeoxyglucose(18F) was 9.0mCi. All scans were performed within a 60 to 90 minute window after injection.

**Figure 3:**
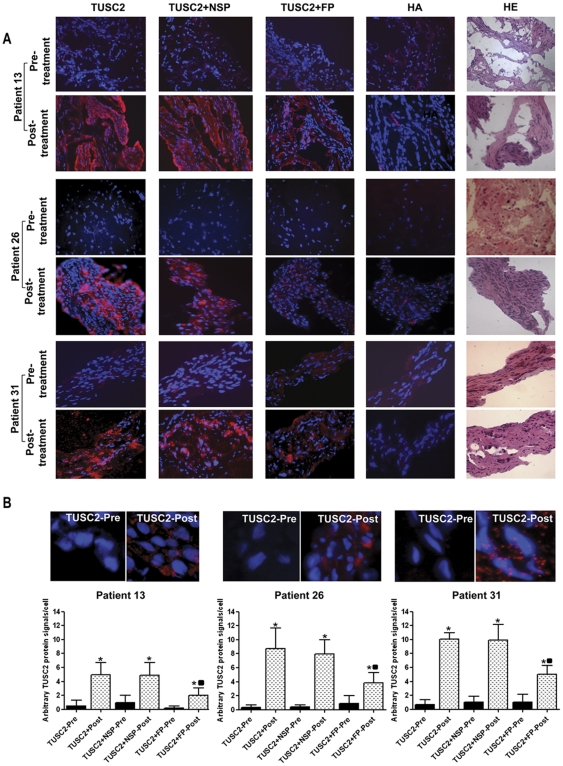
*In situ* Proximity Ligation Assay (PLA) for TUSC2 protein in tumor biopsies (A). A synthetic oligopeptide (GASGSKARGLWPFASAA) derived from the N-terminal amino-acid sequence of the TUSC2 protein was used to develop the anti-TUSC2 polyclonal antibody in rabbits used in this study. Red denotes TUSC2 positivity. DAPI nuclear staining is blue. All panels represent overlays of TUSC2 antibody and DAPI staining. Detailed methods are provided in the Information S1. Pre- and post-treatment biopsies from patients 13, 26, and 31 were tested. Magnification is X40. Panels: (1) anti-TUSC2 antibody; (2) anti-TUSC2 antibody pre-absorbed with non-specific control peptide (NSP); (3) anti-TUSC2 antibody pre-absorbed with TUSC2 peptide (FP); (4) non-specific control antibody; (5) hematoxylin and eosin. Quantitation of PLA signals for pre- and post-treatment samples from patients 13, 26, and 31 (**B**). The anti-TUSC2 antibody was tested under the conditions described in A). The upper panels show PLA signals from the respective patient biopsies as detected by the anti-TUSC2 antibody with 400× magnification. The lower panel presents quantitative comparisons of six independent fields from each biopsy treated under the specified conditions. TUSC2 expression was significantly increased in post-treatment samples compared to pretreatment samples. TUSC2 expression was not significantly altered by anti-TUSC2 antibody pre-absorption with non-specific control peptide (NSP), but was significantly decreased by pre-absorption with TUSC2 peptide (FP). * p<0.05 compared to corresponding pretreatment sample; ▪ p<0.05 compared to post-treatment samples unabsorbed or pre-absorbed with NSP. All comparisons are by two-tailed unpaired Student's t-test assuming equal variances as determined by F test.

Twenty-three patients received two or more doses (Table 2). Five patients achieved stable disease which had a duration of 2.6 to 10.8 months (median 5.0, 95%CI 2.0–7.6) until tumor progression, while all other patients continued to have tumor progression. Two patients had reductions in primary tumor size of 14% and 26%. One patient with stable disease (patient 26) had evidence of a metabolic response on positron emission tomography (PET) imaging and received 12 cycles of therapy. The response was documented with PET scans performed after the second, fourth (Figure 2), and sixth doses, all showing decreased metabolic activity with no changes in size or number of metastases by CT imaging. This patient remains alive on subsequent therapy 14 months after the final treatment with DC-*TUSC2*. Median survival for all patients was 8.3 months (95% CI 6.0–10.5 months) and mean survival time was 13.2 months (95%CI 8.9–17.5 months) with a range of 2 to 21+ months. Two of the five patients with stable disease were in the group which had tumor biopsies. Both patients (Patient 1 with stable disease for six cycles of treatment and Patient 26 as described above) had evidence of transgene expression by RT-PCR. Patient 26 could also be tested for TUSC2 protein expression which was shown to be increased in the post-treatment biopsy (Fig. 3).

### TUSC2 mRNA Expression Detected by RT-PCR

*TUSC2* transgene RNA expression by RT-PCR was not detected in pre-treatment biopsies (Table 3). Five of six post-treatment biopsies showed expression of the *TUSC2* transgene. In a seventh patient (Patient No. 31), TUSC2 mRNA was detected by RT-PCR transgene specific primers included in the qRT Profiler Apoptosis PCR Array and was detected only in the post-treatment sample (see below). Expression was not detected in pre- and post-treatment peripheral blood lymphocytes collected at the time of the biopsies.

**Table 3:** Real Time *RT-PCR* detection of *TUSC2* gene expression in patients receiving DC-*TUSC2*.

Patient Number	Dose (mg/kg)	Site of Tumor Biopsy	Treatment	Tumor *TUSC2* Gene Expression (pg/ug tissue)	Tumor TUSC2 Copy Number (copies/ug tissue)	Lymphocyte *TUSC2* Gene Expression (pg/ul)
1	0.02	Lung	Pre-treatment	0	0	NA^1^
1	0.02	Lung	Post-treatment	2.0×10^−5^±2.20×10^−10^	4.44	NA
7	0.01	Lung	Pre-treatment	0	0	NA
7	0.01	Lung	Post-treatment	3.6×10^−6^±9.1×10^−7^	0.89	NA
13	0.02	Lung	Pretreatment	0	0	NA
13	0.02	Lung	Post-treatment	3.0×10^−5^±1.71×10^−8^	6.22	NA
20	0.06	Liver	Pre-treatment	0	0	0
20	0.06	Liver	Post-treatment	0	0	0
24	0.09	Subcutaneous nodule	Pre-treatment	0	0	0
24	0.09	Subcutaneous nodule	Post-treatment	8.0×10^−6^±2.33×10^−8^	1.90	0
25	0.06	Lung	Pre-treatment	0	0	0
25	0.06	Lung	Post-treatment	4.0×10^−5^±1.66×10^−9^	8.76	0

1 Specimens not available.

### Protein Expression Detected by Proximity Ligation Assay (PLA)

For three patients sufficient tissue was obtained for determination of TUSC2 protein expression by proximity ligation assay (PLA). Anti-TUSC2 antibody was used to detect TUSC2 protein expression in pre- and post-treatment lung tumor biopsies from patients 13, 26, and 31 (Figure 3). The post-treatment biopsies showed intense staining (10 to 25-fold pre-treatment levels) for TUSC2 protein with very low levels of TUSC2 protein detected on the paired pre-treatment biopsies. A non-specific control antibody showed only background staining. Pre-incubation of the TUSC2 antibody with the specific TUSC2 peptide used to immunize for antibody production, but not a non-specific peptide, was able to significantly reduce TUSC2 fluorescence in the post-treatment biopsies.

### Changes in the Apoptosis Pathway Following *TUSC2* Treatment

Significant differences in gene expression were detected by an apoptosis multiplex array assay between pre and post-treatment biopsies from patients 13 and 31. Post-treatment biopsies from both patients showed increased levels of vector specific TUSC2 mRNA not present in the pre-treatment biopsies. Twenty-nine genes of the 82 tested in the apoptosis array were identified by Igenuity Pathway Analysis (IPA, Ingenuity Systems, Inc. Redwood City, CA) to be significantly altered post-treatment in both patients. This subset of genes in the array encompasses the intrinsic apoptosis pathway. The 29 genes in common between the two patients were highly positively correlated with respect to magnitude and direction of change (Pearson correlation coefficient 0.519; p<0.01) The changes in gene expression and canonical apoptosis pathways following DC-*TUSC2* treatment are shown in Figures S3A and S3B.

## Discussion

Loss of heterozygosity in the 3p region is frequently observed in NSCLC. [13] Genes in the 3p21.3 region are associated with cell differentiation, cell proliferation, cell cycle kinetics, signal transduction, ion exchange and transportation, apoptosis, and cell death. [14] These biological activities are directly or indirectly associated with the tumor suppression observed when several 3p21.3 genes (including *TUSC2*) are re-activated in 3p-deficient tumor cells. Forced expression of wt-*TUSC2* in 3p21.3-deficient NSCLC cells significantly suppressed tumor cell growth by inducing apoptosis and altering cell cycle kinetics *in vitro* and *in vivo*, thus providing a strong rationale for gene replacement of *TUSC2* in lung cancer as a therapeutic strategy. [8] While point mutations are rare in the genes at the 3p21.3 locus, [14] there appear to be regulatory mechanisms that can result in further reduction of TUSC2 protein expression in the presence of a single functioning allele. Myristoylation stabilizes the TUSC2 protein and is required for TUSC2-mediated tumor suppressing activity. TUSC2 myristoylation is frequently absent in tumors. When combined with 3p21.3 loss of heterozygosity, loss of myristoylation results in the absence of functional TUSC2 in cancer cells. [15] Noncoding RNAs may also regulate expression of TUSC2. [16]

Nanoparticles have been reported to deliver drugs and siRNA to tumors in humans. [17], [18] The current report is the first to document restoration of tumor suppressor gene function in primary and metastatic cancers following intravenous gene delivery as shown by mRNA and protein expression, alterations in the targeted intrinsic apoptotic pathway, and effects on tumor growth and metabolic activity. A major limitation of some lipid nanoparticle gene delivery formulations is instability *in vivo*, possibly due in part to interactions with serum proteins. [19] Templeton and coworkers first showed that the DC formulation was a more efficient gene delivery system compared to other lipid formulations. [20] Uptake by the liver was markedly reduced compared to other lipid formulations and distribution to organs other than the liver and spleen was increased, with the lung showing the highest level of gene expression. The DC formulation prevents binding of proteins to lipid, thus reducing clearance by the reticuloendothelial system. [21], [22] Cationic lipids are a preferred nanoparticle component for nucleic acid delivery, due to the high efficiency of nucleic acid transfer associated with these lipid formulations. Adding neutral lipids to the cationic lipids increases nanoparticle rigidity and stability. [23] The DC lipoplex formulation was selected for our study because it achieves a balance of low toxicity and efficient nucleic acid transfer in vivo. [20] Due in part to enhanced endocytosis by tumor cells, [24] uptake of these nanoparticles into tumor cells is 10-fold greater than into normal cells, thus imparting a passive targeting property without the need to attach ligands. Using disseminated human cancer cells in mouse xenograft lung metastases, we showed the ability of systemic administration of genes via this DC nanoparticle vector to efficiently deliver therapeutic tumor suppressor genes, to suppress metastasis growth and to prolong survival. [15], [25], [26] An optimized plasmid was developed by using a high-copy number pMB1 plasmid replication origin element, incorporating a mini-CMV promoter (not tumor selective), and removing unnecessary plasmid backbone sequences to keep the plasmid size to a minimum. We also incorporated E1 enhancer elements with the mini-CMV promoter since investigators using recombinant adenoviral vectors have previously noted increased expression of transgenes if the transgene is driven by a CMV promoter that is placed under the influence of an E1 enhancer. [27], [28] The E1 enhancer was derived from the adenovirus serotype 5 E1A transcriptional control region containing repeated core sequences. The E1 enhancer is located between −141 and −305 relative to the E1A cap site at +1. It functions as an enhancer in cis for enhancing the rate of transcription and steady-state levels of E1A mRNAs in virus infected cells. [29]

Posttreatment tumor biopsies 24 hours after intravenous gene therapy administration showed TUSC2 mRNA expression in 7 of the 8 assessable patients and showed elevated levels of protein expression in all 3 patients tested.. Although the number of biopsies was limited due to regulatory constraints, the consistent results across test platforms and high degree of statistical significance suggest these observations are reliable. When mRNA was detectable, the lowest level was seen at the lowest plasmid dose. One patient at a higher dose did not have detectable mRNA at the time point measured, possibly due to endogenous tumor resistance to DC nanoparticle uptake, which we have observed in some lung cancer cell lines (Meng, J., Ji, L., and Roth, J., unpublished observations). Interestingly, the patient who had the metabolic response had a lung carcinoma with neuroendocrine features which expressed very low levels of TUSC2 protein. Prudkin and coworkers observed the lowest levels of TUSC2 in small cell carcinomas(SCLC), which are also neuroendocrine in origin. [11] They also observed loss or reduction of TUSC2 protein in 82% of non-small cell lung cancers (NSCLC). We detected very low levels of TUSC2 protein in pretreatment biopsies from both responding and non-responding patients. This observation and the overall high incidence of TUSC2 loss in lung cancer suggest that loss of TUSC2 protein expression alone may not be a predictive biomarker for *TUSC2* gene therapy.

In this clinical trial, therapy cycles were administered at three week intervals in order to provide adequate observation time for toxicity. Previous pharmacokinetic studies in mice showed circulation in the blood for up to seven days and a t_1/2_ of nine to twelve hours (Barnhart K, *et al*. Systemic apoptotic gene therapy for pancreatic cancer: multi- dose toxicity study of the BikDD nanoparticle. In: *ASGCT 13th Annual Meeting*. Washington, DC; 2010). Alternative schedules for drug administration warrant testing. Using the dose-schedule tested, this tumor suppressor gene therapy was well-tolerated with minimal toxicity. Toxicities may be related to non-specific effects of DC-mediated DNA delivery or off-target effects of gene expression in normal tissues. However, the empty DC particle does not cause toxicity in animals, and uptake of the gene by normal cells in culture does not cause changes in growth characteristics. [8] [30], [31], [32] The low level of side effects after repeated DC administrations is very encouraging suggesting that DC-*TUSC2* may be combined with small molecule targeted drugs at an effective dose.

We observed major changes in gene expression in the intrinsic pro-apoptotic pathway in the tumor following DC-*TUSC2* treatment. Several lines of evidence suggest this is related to expression of TUSC2 in the tumor. Preclinical studies in human lung cancer cell lines identified the intrinsic apoptosis pathway as a mediator of cell death following forced expression of TUSC2. [33], [34] DC complexed with control plasmid DNA did not induce apoptosis in lung cancer cells in preclinical studies. Finally, pre- and post-treatment peripheral blood mononuclear cell intracellular and plasma cytokine levels for cytokines shown to be induced by lipid-DNA complexes in mouse studies (IL-1beta, IL-6, IL-8, IFNgamma, and TNFalpha) were not elevated in patients post-treatment, most likely because of the peri-treatment immunosuppressive regimen, and thus did not contribute to a non-specific immune response (data not shown). The lack of any significant toxicity also strongly mitigates against such a mechanism. In conclusion, we observed for the first time that a functioning TSG can be delivered intravenously to human cancer cells using a nanoparticle vector, express mRNA and protein in cancer cells in the primary tumor and distant metastatic sites, alter relevant pathways in the cancer cell, and mediate anti-tumor activity.

## Supporting Information

Information S1 **Detailed Methods.**
(DOC)

Protocol S1 **Phase I Trial Protocol - Phase I Study of IV DOTAP: Cholesterol-Fus1 in Non-Small-Cell Lung Cancer.**
(PDF)

Figure S1 **Structure and restriction enzyme map of pLJ143/KGB2/FUS1 plasmid vector.** TUSC2 is the symbol approved by the HUGO Gene Nomenclature Committee (HGNC) database for what was previously designated FUS1. In the following, FUS1 and TUSC2 refer to the same gene. These are the original files used for regulatory approval of the clinical trial.
(DOCX)

Figure S2 **Positions and direction of DNA sequencing primers for pLJ143/KGB2/FUS1 vector.** The entire DNA sequence of the plasmid vector is determined by automated DNA sequencing using the DNA Sequencing Core Facility at the M.D. Anderson Cancer Center. The positions and directions of the primers that are used for DNA sequencing are shown. The complete DNA sequence of pLJ143/KGB2/FUS1 plasmid vector are shown in Table 2.
(DOCX)

Figure S3 **Genes which show significant changes in expression for both patients 13 and 31 by IPA biomarker comparison analysis.** **A:** Change in apoptosis pathway mRNAs analyzed in pre and post-treatment biopsy specimens from patients 13 and 31 using SA Apoptosis Signaling Nano-scale PCR Array. Criteria for selection are presented in the Ingenuity Apoptosis Pathway Analysis section of the Information S1. Increased exogenous TUSC2 mRNA expression was detected in the post-treatment biopsy from both patients. **B:** Canonical apoptosis pathway gene expression pertubations following TUSC2-nanoparticle treatment. Canonical apoptosis pathway gene expression pertubations following TUSC2-nanoparticle treatment as detected by SA PRC Array and IPA Analysis. Molecules are represented as nodes, and the biological relationship between two nodes is represented as an edge (Line). The intensity of the node color indicates the degree of up- (red) or down- (green) regulation Nodes are displayed using various shapes that represent the functional class of the gene products. Edges are displayed with various labels that describe the nature of the relationship between the nodes (e.g., P for phosphorylation, T for transcription). The identified nodes indicate perturbation of elements of the intrinsic and extrinsic apoptotic pathways following treatment with DOTAP:chol-TUSC2.
(DOCX)

Table S1 **Dose-Escalation Scheme.**
(DOCX)

Table S2 **Plasmid quality control specifications.**
(DOCX)

Table S3 **DNA sequence of pLJ143/KGB2/FUS1.**
(DOCX)
