# Supplementary material for: Phase I Clinical Trial of Systemically Administered TUSC2(FUS1)-Nanoparticles Mediating Functional Gene Transfer in Humans
Source: PLoS One. 2012 Apr 25;7(4):e34833. doi: 10.1371/journal.pone.0034833 (PMC3338819; doi:10.1371/journal.pone.0034833)
Supplement: Information S1 — Detailed Methods. (DOC) [file pone.0034833.s001.doc]

SUPPORTING INFORMATION S1 – DETAILED METHODS

Study Design and Statistical Analysis

DOTAP:chol-TUSC2 was administered at escalating doses as a 30 minute infusion in a peripheral vein in a total volume of 100 mL of 5% dextrose solution. Patients received DOTAP:chol-TUSC2 every 21 days for up to 6 treatment cycles. After the ninth patient was enrolled, the protocol was amended to require diphenhydramine 50 mg orally or intravenously 30 minutes prior to treatment and dexamethasone 8 mg orally 24 and 12 hours before treatment, 20 mg intravenously 30 minutes prior to treatment, and 8 mg orally 12, 24, and 36 hours after treatment.

Patient Selection

Other eligibility criteria included: Eastern Cooperative Oncology Group (ECOG) performance status ≤ 1; adequate hematologic, hepatic, and renal function; prothrombin time and partial thromboplastin time ≤ 1.25 times the upper limit of normal; left ventricular ejection fraction > 50%; forced expiratory volume in 1 second (FEV1) and diffusing capacity of the lung for carbon monoxide (DLCO) ≥ 40% of predicted; and negative human immunodeficiency virus serology test. Exclusion criteria included: prior gene therapy; brain metastases, unless treated, asymptomatic, and not requiring steroid therapy; chemotherapy within 21 days before enrollment; radiation therapy within 30 days before enrollment; investigational therapies within 30 days before enrollment; active infection requiring antibiotic therapy; myocardial infarction or angina within 6 months before enrollment; and pregnancy or lactation.

**Assessments**

A history and physical examination were performed before every cycle. Adverse events were assessed and laboratory tests performed prior to each cycle and on days 2, 3, and 8. Laboratory tests included a complete blood count with differential, sodium, potassium, chloride, calcium, albumin, total protein, blood urea nitrogen, creatinine, alanine aminotransferase, aspartate aminotransferase, alkaline phosphatase, lactate dehydrogenase, and total bilirubin. Urinalysis and electrocardiograms were obtained prior to each cycle.

**Expression of TUSC2 plasmid in human tumors**

Ectopic expression of the TUSC2 gene in patient biopsy samples was analyzed using a TaqMan based quantitative real time reverse transcriptase-polymerase chain reaction (RT-PCR) (Applied Biosystems, Foster City, CA). RNA was isolated using RNeasy minikit from Qiagen (Valencia, CA) following the manufacturer’s instructions. The fine-needle biopsy tissues that were immediately fixed in RNAlater (Ambion, Austin, TX) were washed once with cold PBS and then the total RNAs were isolated with (reagent and methods). The quality of the purified RNA was analyzed using an Agilent 2100 Nano Bioanalyzer (Agilent Technologies, Santa Clara CA). Reverse transcription was done using a High Capacity cDNA Reverse Transcription Kit (Applied Biosystems, Foster City, CA) with MultiScribe Reverse Transcriptase for two hours at 37^o^C in a thermal cycler according to the manufacturer’s instructions. The PCR reaction was setup with 10 ml of 2X TaqMan gene expression master mix containing the polymerase, buffer and dNTPs, 1 ml of 20X TaqMan gene expression assay solution containing primers, probe, and 5 ml of cDNA template, and 4 ml of sterile distilled water. The primers and probes used were specific to the exogenous TUSC2 transcripts expressed through the plasmid gene expression cassette (Forward primer: 5’ GGA CCT GCA GCC CAA GCT 3’ and Reverse primer: 5’ GCC CAT GTC AAG CCG AAT T 3’, and TaqMan probe: 6-FAM- CGA GCT CGG ATC CAC TAG TCC AGT GTG –TAMRA) to avoid detection of endogenous TUSC2 mRNA. PCR analysis was performed using a 7500 Real-Time PCR System (Applied Biosystems, Foster City, CA) and run with an absolute quantification mode with a standard curve. The DNA amount values were then used for the calculation of TUSC2 copy numbers using the University of Rhode Island’s website [**http://www.uri.edu/research/gsc/resources/cndna.html**](http://www.uri.edu/research/gsc/resources/cndna.html) (URI Genomics & Sequencing Center Calculator for determining the number of copies of a template).

*In situ* Proximity Ligation Assay (PLA) for TUSC2 protein in tumor biopsies

Duolink kits from Olink Biosciences (Uppsala, Sweden) are based on PLA technology and the rolling circle amplification (RCA) reaction wherein a pair of oligonucleotide labeled secondary antibodies (PLA probes) generates a signal only when the two PLA probes have bound in close proximity, either to the same primary antibody or two primary antibodies that have bound to the sample in close proximity. The signal from each detected pair of PLA probes is visualized as an individual fluorescent spot. Signals can be quantified (counted) and assigned to a specific subcellular location based on microscopy images. The rabbit anti-TUSC2 polyclonal antibody used for immunohistochemical staining was raised against a synthetic oligopeptide derived from the TUSC2 NH_2_-terminal amino acid sequence (NH_2_-GASGSKARGLWPFAAC).

The samples are incubated with primary antibodies that bind to the protein(s) to be detected. Secondary antibodies conjugated with oligonucleotides (PLA probe MINUS and PLA probe PLUS) are then added to the reaction and incubated. The ligation solution, consisting of two oligonucleotides and ligase, is added and the oligonucleotides hybridize to the two PLA probes and join to form a closed circle if they are in close proximity. The amplification solution, consisting of nucleotides and fluorescently labeled oligonucleotides, is added together with polymerase. The oligonucleotide arm of one of the PLA probes acts as a primer for a rolling-circle amplification (RCA) reaction using the ligated circle as a template, generating a concatemeric (repeated sequence) product. The fluorescently labeled oligonucleotides will then hybridize to the RCA product. The signal is easily visible as a distinct fluorescent spot that can be analyzed by fluorescence microscopy. In order to detect posttreatment TUSC2 protein expression, we used a single antibody (TUSC2) raised in rabbits and oligo probes (plus and Minus) with rabbit secondary antibodies. *In situ* PLA was performed as per the recommendations of the manufacturer with minor modifications and also including appropriate controls. The experiments were carried out in a blinded setting. Patient biopsy tissues preserved in RNAlater were washed in 50 ml of cold PBS for 30 minutes at 4^o^c before using OCT to prepare frozen blocks to cut slides. The slides with were then fixed with 4% paraformaldehyde and permeabilized with methanol for 20 minutes each. The tissues were blocked for 30 min at 37ºC in a humidified chamber with the blocking buffer provided in the kit and later incubated with anti-rabbit TUSC2 primary antibody overnight at 4ºC. The following day, the primary antibodies were washed and tissues incubated with oligo-linked secondary antibodies (anti-rabbit PLA probes plus and minus). Hybridization, ligation, amplification and detection were then performed according to the manufacturer’s instructions. For non-specific control, rabbit HA tag antibodies were used in the place of TUSC2 antibody. For competition experiments, the synthetic oligopeptide (GASGSKARGLWPFASAA) derived from the N-terminal amino-acid sequence of

the TUSC2 protein that was used to develop anti-TUSC2 polyclonal antibody in rabbits was used ([**Cancer Gene Ther.**](javascript:AL_get(this, 'jour', 'Cancer Gene Ther.');) 2004 Nov; 11 (11): 733-9.

The number of *in situ* proximity ligation signals was counted using the freeware software Blobfinder (<http://www.cb.uu.se/~amin/BlobFinder>). Nuclei were visualized by DAPI staining and used for cell count. The protein expression level was quantified by counting all signals (fluorescent spots) obtained from one image divided by the number of cells in the image, to derive the average signals/cell. Background subtraction was them applied with the pre-treatment samples.

**Antibodies to single and double stranded DNA**

Serum antibodies to single and double stranded DNA were determined by an ELISA assay performed at the Mayo Clinic Department of Laboratory Medicine and Pathology, Rochester, MN. For single stranded DNA antibodies a value of <69 U/ml is considered negative for antibody detection. For double stranded DNA antibodies a value? of <1 is considered negative for antibody detection.

**TUSC2-mediated Apoptosis Pathways using SA Apoptosis Signaling Nano-scale PCR Array**

For gene expression profiling experiments, the total RNAs were isolated from patient fine needle biopsies using Trizol (Invitrogen, Carlsbad, CA) reagent and purified using a RT^2^ qPCR-Grade RNA isolation kit from SA Biosciences (Frederick, MD) according to the manufacturer’s instructions . The purified RNA was then used to synthesize cDNA using RT^2^ Nano PreAmp cDNA Synthesis Kit from SA Biosciences (Frederick, MD). This cDNA kit also involved pre-amplification of the cDNA target templates. The preamplified cDNA was applied onto a RT^2^ Profiler Apoptosis PCR array (SA Biosciences) for qPCR analysis using an ABI 7500 real-time PCR instrument (Applied Biosystems, Foster City, CA) according to the manufacturers’ instructions. The expression level of the mRNA of each gene in the patient after treatment with DOTAP:chol-TUSC2 was normalized using the expression levels of house-keeping genes *B2M, HPRT1, RPL13A, GAPDH, and ACTB*. For data analysis, the comparative C_t_ method was used wherein the relative changes in gene expression were calculated using the ΔΔC_t_ (threshold cycle) method. This method first subtracts the *ct* (threshold cycle number) of the gene-average *ct* of the five housekeeping genes on the array (*B2M HPRT1, RPL13A, GAPDH and ACTB*) to normalize to the RNA amount. Finally, the ΔΔC_t_ was calculated as the difference between the normalized average *ct* of each gene on the array after DOTAP:chol-TUSC2 treatment and the normalized average *ct* of the pre-treatment control sample. This ΔΔC_t_ was then raised to the power of 2 to calculate the relative fold-change of gene expression after-treatment compared to pre-treatment. Genes that differed from pretreatment controls by more than two fold were considered significant and changes of gene expression levels larger than three-fold were shown as a scatter plot.

**Plasmid production**

The first twenty-five patients were treated with a batch produced at the Baylor College of Medicine Center for Cell and Gene Therapy (Houston, TX). Using stock from the original master cell bank, a second batch was produced under GMP conditions at the Beckman Research Institute of the City of Hope (Duarte, CA) because GMP plasmid was no longer produced at the Baylor facility. Both batches met identical quality control specifications (Table S1). No differences in toxicity were noted in the two batches (Table S2). The sequences of plasmid vector are shown in Table S3.

Production of Plasmid DNA (Baylor College of Medicine, Houston, TX)

## Fermentation

A 1 mL vial of the pLJ143 master cell bank stock was aseptically inoculated into 500 mL sterile Terrific Broth with with 1.6% Glycerol  (Teknova: 1.2% Tryptone, 2.4% yeast extract, 1.6% glycerol. 1x phosphate buffer) supplemented with Kanamycin (Sigma) and grown overnight (15-18 hours) at 37°C. This was then used to inoculate 20L of Terrific Broth in a New Brunswick scientific BioFlo IV 4500 fermentor, operating at 37°C, 250-300 rpm, 20-30% dO_2_. Cells are harvested by centrifugation, washed once (1 mL buffer per g wet cell paste) with Alkaline Lysis Solution I(Teknova: 50mM Glucose, 25mM Tris-HCl, pH 8.0, 10mM EDTA, pH 8.0, sterile solution) and frozen at –80°C.

## Lysis and Clarification

The cell pastes were removed from –80°C storage and thawed in a 4°C refrigerator overnight. The cell paste was mixed with Alkaline Lysis Solution I at 8 ml per gram of wet cell paste. The pLJ143 suspension was then mixed 1:1 (v/v) with Alkaline Lysis Solution II (Teknova: 200mM NaOH, 1.0%SDS, sterile solution). After mixing, the material was allowed to lyse at room temperature between 8 and 10 minutes. Two volumes of Alkaline Lysis Solution III (Teknova: 3M potassium acetate, 1.18M formic acid, pH 5.5, sterile solution) were then added with the lysate, and mixed on ice to ensure complete neutralization and precipitation of host cell proteins, genomic host cell DNA and SDS. The neutralized cell lysate was clarified using a bucket centrifuge at 4000 rpm for 30 minutes at 4°C. The supernatant was decanted and clarified through a 1.2 μM PP2 filter.

Purification Strategy by Column Chromatography

This four step purification process does not require RNase enzyme, organic solvents, detergents, precipitants or animal derived components. The entire process is controlled with an Aekta Purifier (Amersham Bioscience) and Unicorn Software (Amersham Bioscience). All columns and packing material are from Amersham Bioscience. All column preparation and storage is as follows:

*CIP:* 0.5 M NaOH, 25°C for I hour contact time

*Depyrogenation:* 100 ppm sodium hypochlorite pH 10, then 0.1-.5N NaOH, pH13

*Storage:* 20% ethanol (aqueous solution)

*Preparation for* Use (Sanitization): Cell culture grade water (US Pharmacopia), then primed with applicable buffer

*Step 1:Concentration Using Hollow Fiber Filter (HFF)*

The clarified lysate was first concentrated approximately 10-fold and equilibrated with using a 300,000 kDal nominal molecular weight cut-off (NMWCO) A/G Technology hollow fiber filter (HFF). The HFF was flushed with 4-L of Alkaline Lysis Solution III (3M potassium acetate, 1.18M formic acid, pH 5.5, sterile solution) and pooled with the concentrated lysate. A final volume of approximately 2-L is recovered, filtered with a 0.45μm filter, and stored at 4°C until the next step.

*Step 2:* *Size Exclusion*

RNA removal and buffer exchange by group separation using Sepharose 6 Fast Flow with BPG size exclusion column . UF Concentrate is applied to the column in batches of 0.3 column volumes (CV) to change the buffer to Buffer A. Simultaneously this procedure also removes RNA and other contaminants. The void fractions are stored at 4^0^C and then pooled for the next step.

Buffer A: 2M (NH_4_)SO_4_, 10 mM EDTA, 100 mM Tris_HCl, pH 7.0

*Step 3: Selective capture of supercoiled plasmid DNA by thiophilic aromatic adsorption chromatography.*

Supercoiled plasmid DNA is separated from open circular plasmid DNA and remaining contaminants such as residual genomic DNA and RNA. The pooled void fraction (from Step 2) is subsequently applied on the XK50 Affinity column packed with PlasmidSelect and equilibrated in the same buffer A. The column is washed and supercoiled plasmid DNA is eluted with Buffer B. Fractions are stored at 4^0^C prior to next step. Fractions are pooled for step 4. then diluted with four volumes of water for the next step.

Buffer A: 2M (NH_4_)SO_4_, 10 mM EDTA, 100 mM Tris_HCl, pH 7.0

Buffer B: 1.4M NaCl, 2.0 M (NH_4_)SO_4_,10 mM EDTA,100 mMTris-HCl, pH7.0.

#### *Step 4: Polishing and concentration with SOURCE 30Q*

Endotoxins are further removed and at the same time, the supercoiled plasmid DNA preparation is concentrated by ion exchange chromatography.The fraction (from step 3) containing supercoiled plasmid DNA is diluted with 4 volumes of pharmaceutical grade water and loaded on a XK26 ion exchange column packed with SOURCE 30Q. The column is equilibrated Buffer C and eluted with a linear gradient, Buffer D. The fractions are then pooled and filtered through a 0.22μm filter.

Buffer C: 0.4 M NaCl, 10 mM EDTA, 100 mM Tris_HCl, pH 7.0

Buffer D: 1.0 M NaCl, 10 mM EDTA, 100 mM Tris_HCl, pH 7.0,

PlasmidSelect is the key protocol component since it interacts differentially with nucleic acids by thiophilic aromatic adsorption in the presence of water structuring salts. This enables the topoisomere_selective purification of native supercoiled plasmid DNA and removal of damaged, nicked or open circular DNA by simple adjustment of chromatographic conditions. A group separation for removal of RNA prior to application on the column optimizes the capacity of PlasmidSelect for binding of the supercoiled form of plasmids. Furthermore, group separation with Sepharose 6 Fast Flow greatly reduces the risk of precipitation during addition of ammonium sulfate and limits the variation in initial salt concentration that can influence selectivity, thus giving the process considerable robustness.

#### Precipitation

Ethanol precipitation was used to concentrate the pLJ143 to 5mg/ml. A 3.0M sterile NaCl solution was used to increase the NaCl concentration of the pLJ143 solution to 0.15M. Ethanol was added into the pLJ143 solution in a 2:1 ratio to give a final ethanol concentration of 67%. The pLJ143 suspension was stored at –20°C overnight to allow for complete precipitation. The next morning, the pLJ143 was recovered by centrifugation. The pLJ143 pellets were further washed with 70% ethanol and allowed to air dry aseptically in a laminar flow hood for approximately one hour. Dried pellets were frozen at -80°C until purification of wet paste from all fermentation runs was complete. The pLJ143 was then reconstituted at 5mg/ml in sterile endotoxin, RNAse, DNAse free water. All work is performed in a class 100 biosafety cabinet.

### **Vialing**

Product was filled into 1.2 ml crimp cap glass vials with semi-automated dispensing pipette in a class 100 biosafety cabinet. The fill volume is either 0.3 ml, 0.5 ml, or 1.0 ml. The target plasmid DNA concentration was 5 mg/mL. The final product is stored at -80°C.
