# Supplementary material for: Phase I Clinical Trial of Systemically Administered TUSC2(FUS1)-Nanoparticles Mediating Functional Gene Transfer in Humans
Source: PLoS One. 2012 Apr 25;7(4):e34833. doi: 10.1371/journal.pone.0034833 (PMC3338819; doi:10.1371/journal.pone.0034833)
Supplement: Protocol S1 — Phase I Trial Protocol - Phase I Study of IV DOTAP: Cholesterol-Fus1 in Non-Small-Cell Lung Cancer. (PDF) [file pone.0034833.s002.pdf]

TITLE Phase I Study Of Intravenous DOTAP:Cholesterol-*Fus1* Liposome Complex (DOTAP:Chol-*fus1*) In Patients With Advanced Non-Small Cell Lung Cancer (NSCLC) Previously Treated With Chemotherapy

1.0. Objectives
Primary Objectives

1.1 Assess the toxicity of DOTAP:Cholesterol-fus1 Liposome Complex (DOTAP:Chol-*fus1*) administered intravenously.

1.2 To determine the maximal tolerated dose and recommended phase II dose of DOTAP:Chol-*fus1* administered intravenously.

Secondary Objectives

1.3 Assess the expression of *fus1* following intravenous delivery of DOTAP:Chol-*fus1* in tumor and normal bronchial epithelial cell biopsies.

1.4 Assess any anti-cancer activity for DOTAP:Chol-*fus1*.

2.0. Background

In the United States lung cancer is the most lethal cancer in both men and women with more than 150,000 deaths annually [1]. The majority of lung cancer patients present with locoregionally advanced stage III disease or with metastases to distant organs (stage IV). Overall survival for lung cancer has not changed over the past 25 years.

As understanding of the molecular and genetic mechanisms of oncogenesis has increased, the focus of cancer treatment has shifted in recent years from the tissue to the genetic level [2]. Mutations in two major classes of genes—oncogenes and tumor suppressor genes—have been implicated in the oncogenic process. Oncogenes are normally involved in functions such as signal transduction and transcription, whereas some tumor suppressor genes play a role in governing proliferation by regulating transcription. Functional alterations due to mutations in either class of gene may result in the abnormal and uncontrolled growth patterns characteristic of tumors. Oncogene mutations appear to be dominant, while tumor suppressor genes appear to require homozygous deletion or mutation for inactivation.

A region of tumor suppressor genes have been identified on chromosome 3p, specifically located at 3p21.3 in a 450 kb region (and potentially very small 30 kb region) defined by homozygous deletions in lung cancers. Genes in this region are of fundamental importance in the pathogenesis of many human cancers including lung cancer where it is likely to be involved in >50% of all cases Since 3p21 allele loss is the earliest lung cancer genetic abnormality so far detected, occurring in hyperplastic lesions, genes in this region are likely to be the "gatekeepers" for lung cancer pathogenesis. Lung cancer cells harbor mutations in multiple known and

suspected dominant and recessive oncogenes. These include known recessive oncogenes (rb, p53) and putative recessive oncogenes at chromosome regions 3p21, 3p24-25, 3p12-13, 3p14, 5q, 6p, 8p, 9p (p16ink4/MTS1), 11p13, 11p15 as well as other sites [3,4]. The data supporting the existence of such recessive oncogenes comes from cytogenetic and allelotyping studies of fresh tumors and tumor cell lines showing tumor cell allele loss at multiple sites and thus suggesting the presence of mutations in recessive oncogenes in the remaining allele. In fact, as many as 10-20 allele loss changes may occur per clinically evident lung cancer. In all of these studies, changes on the short arm of chromosome 3 (3p) stand out. Cytogenetic and allele loss studies have shown changes in ~90% of small cell lung cancers (SCLC) and >50% of non-small cell lung cancers (NSCLC) [5-14]. In addition, similar 3p changes have been seen in several other human cancers such as renal, breast, head and neck, mesothelioma, ovary, and uterine cervical cancer [15-24]. Recent studies of preneoplastic lesions suggest 3p loss of heterozygosity (LOH) occurs at the earliest stages (hyperplasia) of pathogenesis developing prior to 9p, rb, p53, and ras mutations [25-27]. Cell hybrid and microcell chromosome 3 transfer studies demonstrate the ability of human chromosome 3 genes to suppress malignancy in human lung (one example in a bronchoalveolar carcinoma cell line), renal, and ovarian cancer cell lines and mouse A9 fibrosarcoma cells [28-33].

Although fewer NSCLCs than SCLCs have abnormalities in the 3p21 region, about 50% have LOH in this region and are thus expected to be affected by the same tumor suppressor gene. Dr. John Minna has been analyzing this region, and tests with a variety of other probes, knowledge of the pulse field map, and information from the cosmid contig allowed him to size the the homozygous region of this deletion at ~750kb. Dr. Minna's group isolated and tested a large number of new probes from the LC 3p21.3del-1 against the lung cancer cell line panel of 120 DNAs. This screen discovered a previously undetected homozygous deletion occurring in the SCLC cell line, H524 which mapped to the 330 kb NotI fragment and this deletion was calculated to be only ~30 kb in length. The fusion breakpoints of the H524 deletion were isolated as a genomic clone from H524 DNA and used to isolate two different cDNAs that had not been previously identified in the Genebank, 3p21.3gfus1 and 3p21.3gfus2 (see below).

Unexpectedly, there are nine genes either disrupted or immediately flanking the 30 kb H524 homozygous deletion (3p21gB*, 3p21g123F2, 3p21gfus1, 3p21gluca2, 3p21gluca1, 3p21gfus2, 3p21gluca3, 3p21gskm15, 3p21ghsemA1

Our laboratory has constructed adenoviral expression vectors for each of these genes to test for tumor suppressor function. We found that 3p21gfus1(fus 1) showed the most potent suppression of tumor cell growth through induction of apoptosis.

3.0 Drug Information:

Our collaborator, Dr. John Minna, has been analyzing this region. Tests with a variety of probes, knowledge of the pulse field map, and information from the cosmid contig allowed him to size the homozygous region encompassing this deletion at ~750kb . Dr. Minna's group isolated and tested a large number of new probes from the LC 3p21.3del-1 against a lung cancer cell line panel of 120 DNAs. This screen discovered a previously undetected homozygous deletion occurring in the SCLC cell line H524 which mapped to

the 330 kb *NotI* fragment, and this deletion was calculated to be only ~30 kb in length. The fusion breakpoints of the H524 deletion were isolated as a genomic clone from H524 DNA and used to isolate two different cDNAs that had not been previously identified in the Genebank, *3p21.3gfus1* and *3p21.3gfus2*. Unexpectedly, there are nine genes either disrupted or immediately flanking the 30 kb H524 homozygous deletion (3p21gB*, 3p21g123F2, 3p21gfus1, 3p21gluca2, 3p21gluca1, 3p21gfus2, 3p21gluca3, 3p21gskm15, 3p21ghsemA1). Our laboratory has constructed adenoviral expression vectors for each of these genes to test for tumor suppressor function. We found that *3p21.3gfus1*(*fus1*) showed the most potent suppression of tumor cell growth through induction of apoptosis (Figure 1A-C). Although other genes in this region showed tumor suppressor activity, *fus1* consistently showed the highest level in vitro and in vivo, and the small size of its cDNA makes it easier to manipulate in expression plasmids relative to the other genes which are larger.

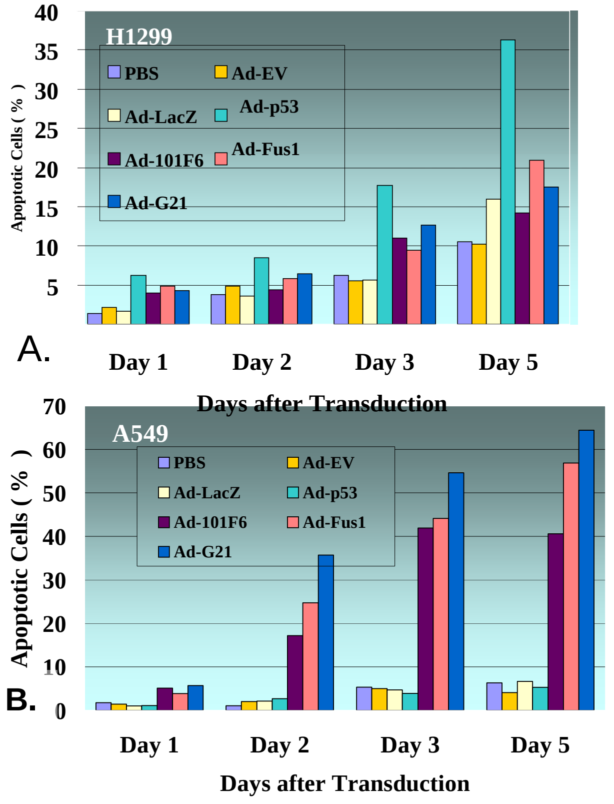

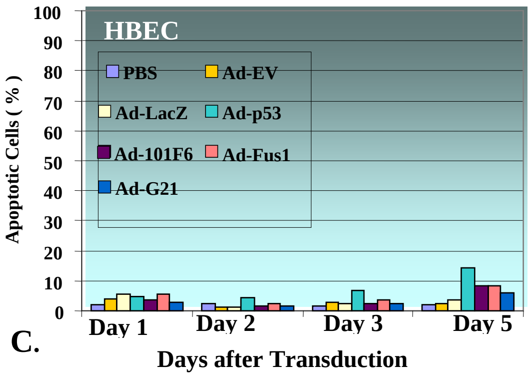

Figure 1: Effects of expression of fus1, 101F6, and G21 expressed in adenoviral vectors I H1299 (A), and A549 (B) human lung cancer cells and human bronchial epithelial cells (C). The apoptotic fraction was measured by FACS using TUNEL reaction with FITC-labeled dUTP. Ad-p53 was used as a positive control and Ad-EV, an E1 deleted adenovirus was used as a negative control.

The precise function of this gene is not yet known. However, it causes a high level of apoptosis in human non-small cell lung cancer cells but not normal human bronchial epithelial cells and thus represents a significant target for therapeutic and prevention strategies for lung cancer. As 3p21 LOH is an early event in lung carcinogenesis, Fus 1 has potential utility even in the earliest stages of cancer and may be a useful target for prevention interventions. Restoration of tumor suppressor gene expression is feasible in lung cancer. Phase I studies using an adenoviral vector expressing the normal tumor suppressor gene p53 (Ad-p53) in the treatment regional cancer,

including squamous cell carcinoma of the head and neck and non-small cell cancer of the lung have indicated that Ad-p53 can be given repetitively with safety and can mediate regression of large tumors.[34,35] However, current viral vectors are limited to intratumoral administration which does not have an effect on pre-existing metastases. Thus development of vectors for **systemic gene replacement** (intravenous administration to treat metastases) could have a significant impact on lung cancer mortality. The most widely used vehicle for the delivery of gene therapy agents is the adenovirus. Adenoviral gene therapy strategies, however, are limited by the potential for patient immune response, a possible inability for repeat administration of viral vectors, a difficulty in generating high viral titers, and the potential of infectious virus production. Lipid based gene delivery systems provide an alternative gene therapy vehicle that is devoid of these problems.

Improved Liposome Complex Mediated Gene Transfer:

Lipid based non-viral formulations provide an alternative to adenoviral gene therapies. Although many cell culture studies have documented lipid based non-viral gene transfer, systemic gene delivery via lipid based formulations has been limited. A major limitation of non-viral lipid based gene delivery is the toxicity of the cationic lipids that comprise the non-viral delivery vehicle. The *in vivo* toxicity of liposomes partially explains the discrepancy between *in vitro* and *in vivo* gene transfer results. Another factor contributing to this contradictory data is the difference in liposome stability in the presence or absence of serum proteins. The interaction between liposomes and serum proteins has a dramatic impact on the stability characteristics of liposomes [36]. Cationic liposomes attract and bind negatively charged serum proteins. Liposomes coated by serum proteins are either dissolved or taken up by macrophages leading to their removal from circulation. Current *in vivo* liposomal delivery methods use subcutaneous, intradermal, intratumoral, or intracranial injection to avoid the toxicity and stability problems associated with cationic lipids in the circulation. The interaction of liposomes and plasma proteins is responsible for the disparity between the efficiency of *in vitro* [37] and *in vivo* gene transfer [38-45]. Lipid based gene transfer vehicles can be produced from various lipids or lipid mixtures that can vary in charge, fluidity, and packing geometry. Cationic lipids are a preferred component for nucleic acid delivery, due to the high efficiency of nucleic acid transfer associated with these lipid formulations. Often, cationic lipids are mixed with neutral lipids to increase the rigidity and stability of the liposome. However, some cationic lipids are toxic when administered *in vivo*. This may be related to the ability of bacterial DNA to stimulate cytokine production resulting in an accumulation of NK [46]. The DOTAP:cholesterol lipoplex is currently the best lipid formulation in regards to balancing toxicity and *in vivo* nucleic acid transfer efficiencies. In previous studies we demonstrated the ability of improved extruded DOTAP:Cholesterol liposome to efficiently deliver therapeutic tumor suppressor genes to experimental disseminated metastases. Metastatic tumor growth is suppressed and survival is prolonged.

Extruded DOTAP:Cholesterol liposomes were compared to non-extruded DOTAP:Cholesterol liposomes and another conventional liposome formulation (DOTAP:DOPE) in a therapeutic xenograft model of human lung metastasis using the H1299 and A549 human lung cancer cells. The extrusion process forms a lipid bilayer which encapsulates the DNA, protecting it from nuclease degradation. Analysis of lungs from p53 null H1299 lung tumor bearing SCID/beige

mice revealed a significant reduction ($p<0.001$) in the number of experimental metastases in mice following tail vein injection of extruded DOTAP:Cholesterol-p53 complex when compared to all control groups.

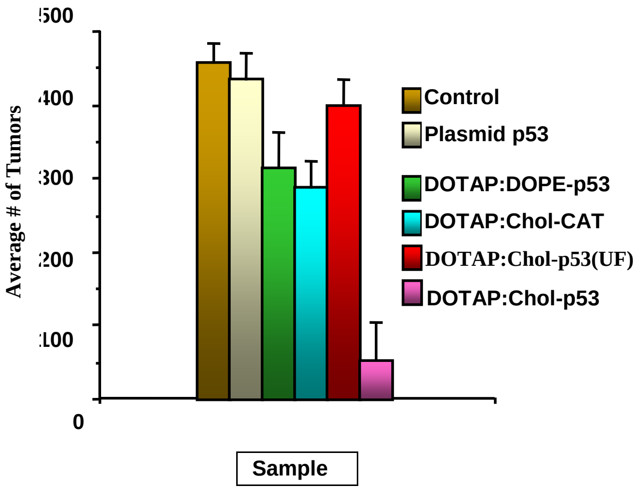

Figure 2: SCID/beige Mice receiving extrudedDOTAP:Cholesterol-CAT complex (group 3) or DOTAP:DOPE-p53 complex (group 4) demonstrated minimal inhibition of H1299 lung colonies when compared to mice receiving no treatment (group 1) or plasmid DNA (group2). No inhibitory effect was observed in mice receiving non-extruded DOTAP:Cholesterol p53 DNA complex (group 5). Mice in group 6 treated with DOTAP:Chol-p53 showed a significant reduction in metastases compared to the other groups ($p<0.001$).

(Figure 2). To determine if the observed p53 tumor inhibitory effect was restricted to only p53 mutated or null tumors or could also be used to treat p53 wild type tumors, we used A549 cells which are homozygously wildtype for the p53 gene and also form lung metatstases following tail vein injection in *nu/nu* mice. A significant reduction in the number of metastases was observed in mice receiving extruded DOTAP:Cholesterol –p53 DNA complex ($p<0.001$) when compared to control groups. Analysis of lung metastases by TUNEL staining showed that apoptosis was occurring, and intravenous injection of the LacZ marker expression plasmid showed high expression levels in metastases (data not shown). Survival following intravenous DOTAP:Chol-p53 complex treatment in mice with disseminated human lung cancer was studied (Figure 3). In this model SCID mice injected intravenously with H1299 cells develop metastases in multiple organs including lung, liver, adrenal, and bone. H1299 lung tumor bearing mice were divided into four groups and treated as follows: group 1 received no treatment, group 2 received p53 plasmid DNA, group 3 received DOTAP: Chol-CAT complex and group 4 received DOTAP:Chol-p53 complex daily for a total of six treatments. All mice from group 1, group 2 and group 3 died from tumor burden between days 30 and 81 after tumor cell injection (mean survival = 38 days in group 1,40 days in group 2 and 41 days in group 3). In contrast mice treated with DOTAP:Cholesterol-p53 DNA complex (group 4) survived substantially longer (median survival = 65 days; $p<0.003$) and 33% of mice remained alive at the end of the experiment on day 150.

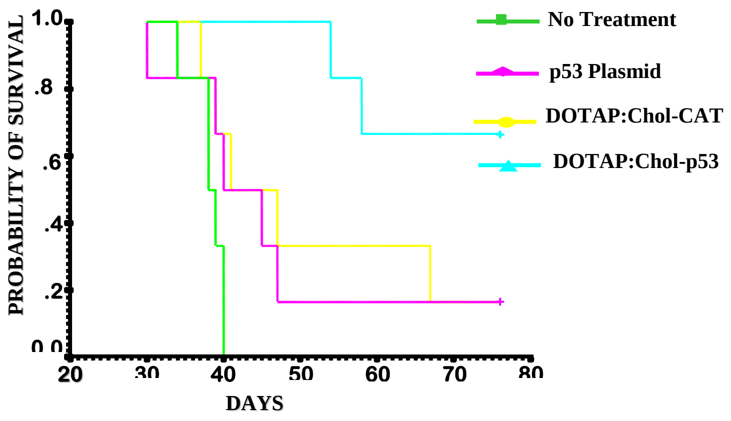

Figure 3: Actuarial survival curves for mice with H1299 cells injected in the tail vein followed by six tail vein injections of either saline, p53 plasmid, DOTAP:Chol-CAT complex, or DOTAP:Chol-p53. Survival for the DOTAP:Chol-p53 group was significantly prolonged compared to the other groups (p<.003)

The results from the preclinical studies of intravenous delivery of tumor suppressor genes complexed to DOTAP:Chol are compelling and justify a clinical trial to assess toxicity as a prelude to possible efficacy studies. The fus 1 gene has been selected because of its high degree of selective apoptosis for lung cancer cells compared to normal bronchial epithelial cells, its ability to completely inhibit the growth of subcutaneous tumors with intratumoral injection, and its ability to mediate a reduction in lung experimental metastases of >80% which is comparable to p53 (Figures 4-7). It is likely that this gene will be deleted in the early stages of lung carcinogenesis thus making it an attractive target for all stages of disease.

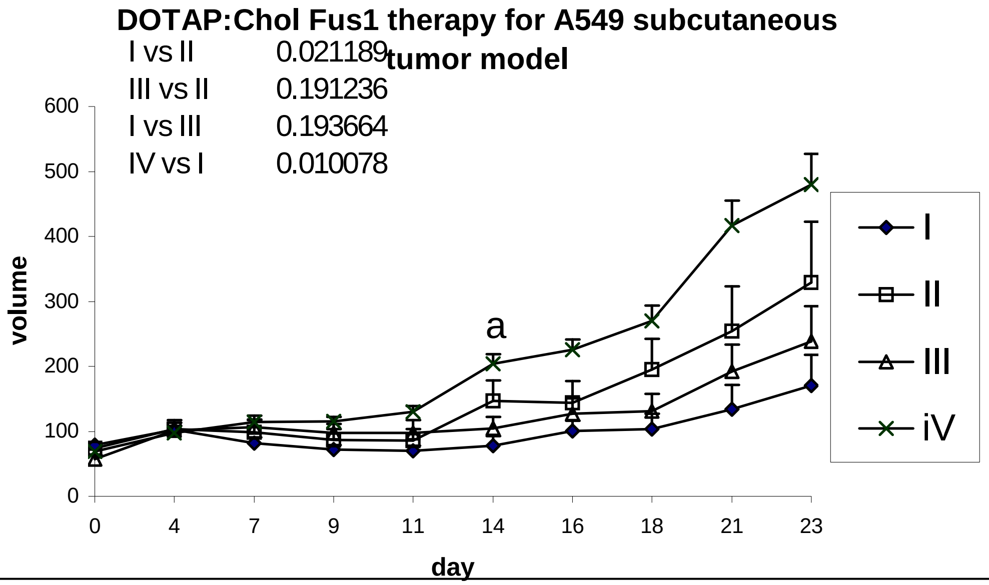

Fig. 4: When the tumor reached 5 to 10 mm in diameter at 2 weeks after tumor inoculation, the tumor was injected with DOTAP:Chol Fus1 (100µg DNA) (I), DOTAP:Chol CAT (100µg DNA) (II), DOTAP:Chol p53 (100µg DNA) (III), all in 200 µl of PBS and 200 µl of PBS as a mock control (IV) for three times per week for a total of six doses. Results were reported as the mean ± SD in 10 mice for each treatment group. Tumor volumes were normalized by the percentage increase of tumor size after treatment relative to those at the beginning of the treatment in each group. Mean tumor volumes ± SE from these experiments are shown. ANOVA was performed to determine statistical significance between each treatment group using a Statistica software (StatSoft Inc.) and $P \leq 0.05$ was considered significant. Fus 1 vs. CAT p<0.05; p53 vs. CAT not significant; FUS 1 vs. p53 not significant; FUS 1 vs. PBS p<.05; PBS vs CAT not significant.

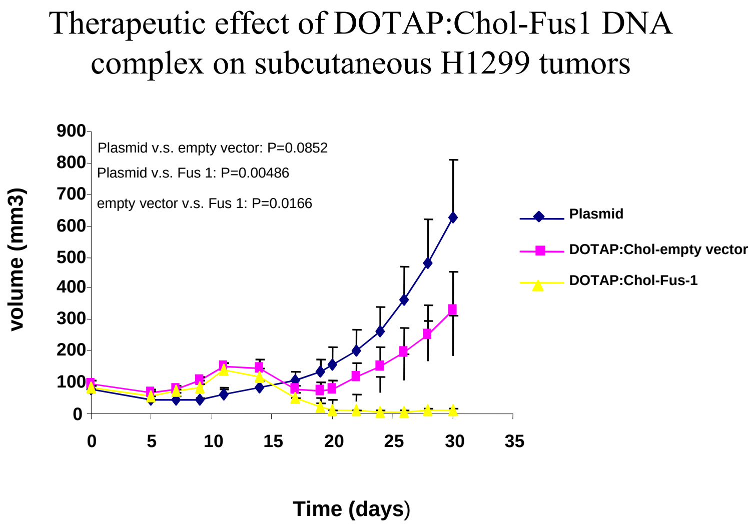

Figure 5: Therapeutic effect of Fus-1 complexed to DOTAP:Chol. liposome treatment on subcutaneous human lung tumor xenografts. Human non small cell lung carcinoma cells (H1299) were injected subcutaneously ($5x10^6$ cells) on the right posterior flank. Animals were randomized into three groups (n=8 / group) and were treated daily for a total of six doses intratumoral doses (100 ug DNA/dose) as follows: Fus-1 plasmid DNA , DOTAP:Chol:empty vector DNA complex, and DOTAP:Chol:Fus1 DNA complex. Tumors were measured using calipers and each time point represents the average tumor volume for each group.

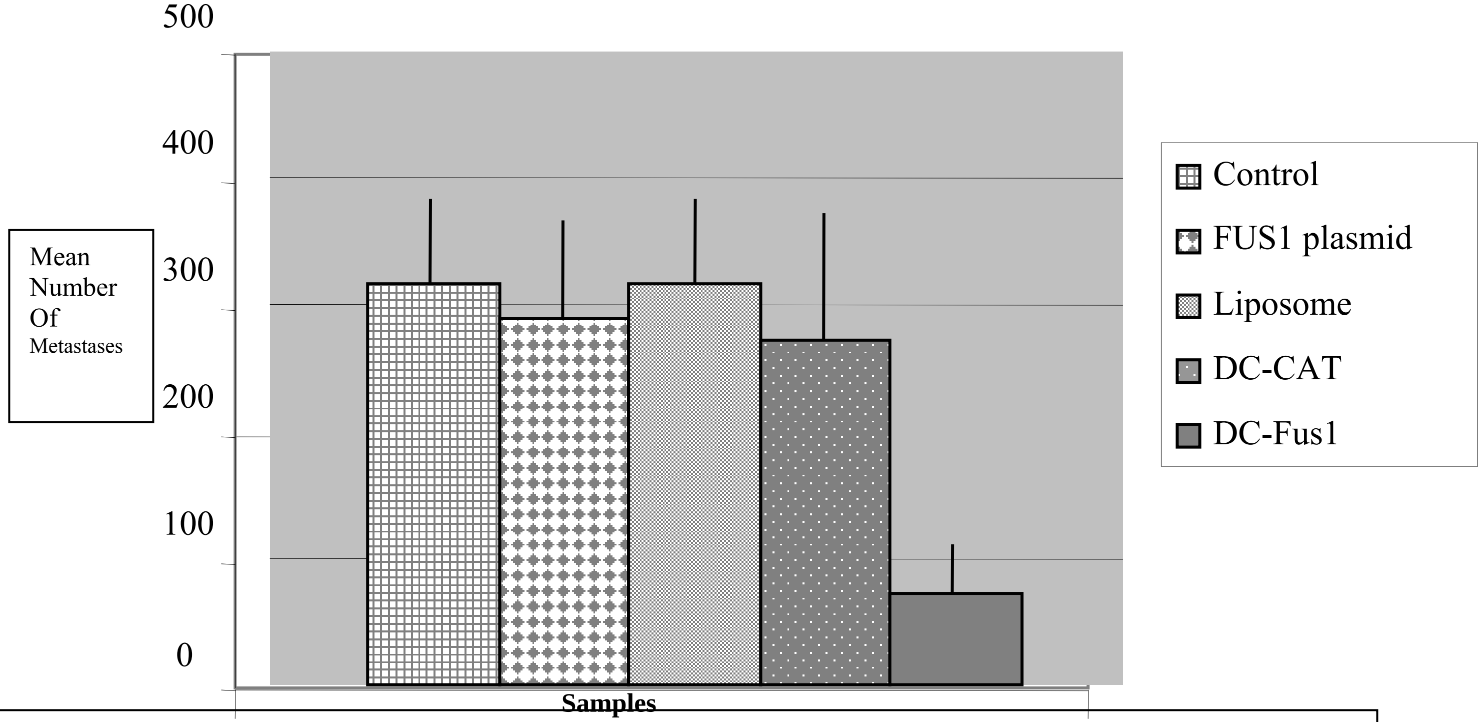

Fig. 6: Female athymic nude mice were injected intravenously with A549 tumor cells (1x106 cells/animal) via tail vein in a total volume of 100 ul. Six-days later, animals were randomly divided into five groups (n=5/group) and treated as follows: Group 1, received no treatment; Group 2 received plasmid DNA; Group 3 received liposome alone; Group 4 received DOTAP:Chol-CAT complex; Group 5 received DOTAP:Chol-Fus1; all animals were treated with 50 ug of plasmid DNA encapsulated in DOTAP:Chol:liposome in a total volume of 100 ul. Animals were treated daily for a total of six doses. Three weeks after the last treatment, animals were euthanized by CO2 inhalation and the trachea exposed. India Ink was injected into the trachea, and lungs were harvested and fixed in Fekete's solution. Lungs were observed under a dissecting microscope and the number of lung colonies counted. Animals from group 5 showed a significant reduction in the number of lung tumor colonies.when compared to animals from groups 1,2,3 and 4 (p<.;05).

Fig. 7 DOTAP:Chol-Fus-1 treatment increases survival of animals with experimental lung metastasis. Experimental lung tumors were established by injecting A549 cells (10^6) via tail vein in female nude mice. Six-days later animals were divided into groups (n=5/group) and treated as follows: treated with PBS, treated with Fus-1 plasmid alone (10 ug), treated with DOTAP:Chol alone, treated with DOTAP:Chol-CAT complex (10ug DNA), and treated with DOTAP:Chol-Fus-1 complex (10 ug DNA). Animals were treated daily for a total of six doses (10 ug DNA/dose) by injecting via tail vein. Animals were monitored daily to assess for mobidity and mortality and survival determined by the. A significant (p=0.01) increase in survival (Kaplan-Meier test) was observed in animals treated with DOTAP:Chol-Fus1 complex than in animals from control groups.

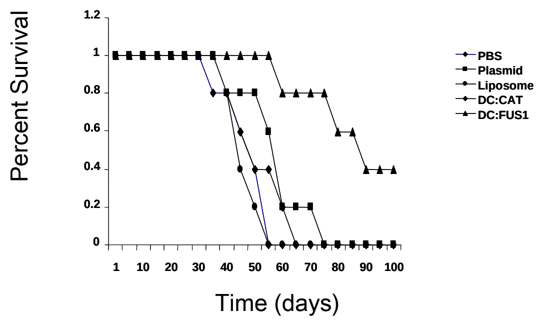

Preclinical Toxicity Studies in Mice

The mouse LD10 for a single intravenous dose of DOTAP:Cholesterol-Fus1 liposome complex was determined from a series of experiments. For each experiment, C3H strain mice (4 to 6 weeks old, estimated total blood volume 1 ml) were injected over a period of approximately 3 minutes. The doses ranged from 50 to 150 mcg of DOTAP:Cholesterol-Fus1 liposome complex, and the total injection volume ranged from 100 to 300 microliters. The results of the dose-escalation study in mice are summarized below:

DOTAP:Chol-Fus1 Dose (micrograms)	Total number of mice	Injection volume (microliters)	Number of deaths (%)
50	8	100	0
60	8	120	0
70	8	140	0
80	8	160	0
90	8	180	0
**100**	**23**	**200**	**2 (8.6%)**
110	18	220	0
120	18	240	1 (5.6%)
130	18	260	7 (39%)
150	23	300	7 (30%)

The **LD10** for a single intravenous injection in mice was conservatively estimated to be **100 micrograms**. Of significance, the drug was infused over approximately 3 minutes, and the injection volumes ranged from 100 to 300 microliters, or the equivalent of 10 to 30% of the animals' total blood volume. This rapid rate of infusion would never be used in humans, and the relationship of the rapid infusion rate to the observed animal toxicity remains unclear. Autopsies were obtained on all animals that died secondary to acute toxicity. Pathological examination of the brain, heart, lungs, spleen, liver, gastrointestinal tract, and kidneys were performed by an attending veterinary pathologist. The pathology findings are summarized below:

Dose (micrograms)	Number of Autopsies	Pathology Findings (# of animals)
100	2	Lymphoid tissue and spleen, necrosis, apoptosis, and atrophy, moderate (2)<br>Multifocal liver degeneration and necrosis, mild (1)<br>Acute liver necrosis, mild (1)
120	1	Lymphoid tissue, spleen, and GALT necrosis, apoptosis, and atrophy, moderate (1)<br>Acute liver necrosis, moderate (1)<br>Malignant lymphoma, kidney (1)<br>Glomerulonephritis (1)

130	7	Lymphoid tissue and spleen, necrosis, apoptosis, and atrophy, mild (1), moderate (6)<br>Acute liver necrosis, mild (3), moderate (3), severe (1)<br>Multifocal myocardial degeneration, necrosis, and mineralization, moderate (2), severe (1)<br>Acute tubular necrosis, kidney, minimal (1)<br>Lung granuloma/foreign bodies (1)<br>Intestinal crypt epithelial acute necrosis, mild (1)
150	7	Lymphoid tissue and spleen, necrosis, apoptosis, and atrophy, mild (3), moderate (4)<br>Acute liver necrosis, mild (4), moderate (1), severe (2)<br>Multifocal myocardial degeneration, necrosis, and mineralization, mild (1), moderate (2)<br>Acute tubular necrosis, kidney, mild (1)<br>Multiple subacute to chronic kidney infarcts (1)<br>Spleen red pulp myeloid hyperplasia (1)<br>Spleen sinus histiocyte marked hyperplasia (1)<br>Intestinal crypt epithelial acute necrosis, mild (1)

Note: Multifocal myocardial degeneration, necrosis, and mineralization are most likely incidental findings observed in control C3H mice (ref. Vargas, KJ, Stephens, LC, Clifford, CB, et al. Dystrophic Cardiac Calcinosis in C3H/HeN Mice. *Lab Anim Sci*, **46**:572-575, 1996.)
Minimal = 1+, 5-10%; Mild = 2+, 10-20%; Moderate = 3+, 20-50%: Severe = 4+, >50%

GLP Toxicology Studies

The objective of this study was to determine single dose toxicology of DOTAP:Chol/*fus1* in preparation for Phase I studies. The non-toxic dose and dose-limiting toxicity for C3H/HeNCR mice were determined. The study contained three control groups: D5W (vehicle), 4 mM DOTAP:Chol (highest dose of lipid), and 70 μg DNA (highest dose of *fus1* plasmid).The study also contained three experimental groups: 70 μg DNA,DOTAP:Chol, 40 μg DNA,DOTAP:Chol and 10 μg DNA,DOTAP:Chol. Each group contained 15 mice (8 female and 7 male). Acute (0-72 hours), subacute (14 days) and chronic (6 weeks) toxicity were evaluated. At 3 and 14 days and at 6 weeks, five mice per group were euthanized. For each mouse, an attempt was made to collect urine for analysis for CBC and serum chemistries. Necropsies were performed and histopathological analysis done on all mice, including those that died during the study. This study was conducted in an AAALAC accredited facility (2000).

All mice in the three control groups (**D5W, 4mM DOTAP:Chol, and 70 micrograms DNA alone**) and in the experimental group receiving **10 micrograms DNA, DOTAP:Chol** were observed to be normal at all observation time points.

Mice in the experimental group receiving **40 micrograms DNA, DOTAP:Chol** appeared normal at the end of the 4 hours post-injection observation period. When observed later that day at approximately 7 hours post injection 14/15 mice were squinting and appeared to be

lethargic. One female mouse was very weak, trembling and sat hunched with her eyes closed. She was euthanized and sent to necropsy at that time. On day one post injection (PI), all mice had decreased activity levels and the eyes appeared to be swollen. On day two PI, all mice appeared to have returned to normal activity levels and general appearance. One female mouse had an area of necrosis involving approximately 20% of one pinna at this time point, but otherwise appeared normal. The damaged pinna was interpreted to be the result of trauma. All mice were thereafter normal at all observation time points. In summary, one female mouse became moribund on day zero and was euthanized.

Mice in the experimental group receiving **70 micrograms DNA, DOTAP:Chol** appeared normal at the end of the 4 hours post-injection observation period. When observed later that day at approximately 7 hours post injection, all mice were squinting and appeared to be lethargic. On day one PI, one female mouse died. Three male mice and one female mouse were found to be moribund and were euthanized and necropsied. One female mouse was reported to have a swollen face. This mouse and the remaining mice in the group all appeared to have decreased activity levels and abnormal appearance at day one PI. On day two PI, the female mouse that had the swollen face on day one PI was found to be moribund and was euthanized and necropsied. Another female mouse was found dead on day two PI. The remaining mice had decreased or slightly decreased activity levels and some were squinting. On day three PI, 2/8 remaining mice appeared normal, while 6/8 still had decreased activity levels and abnormal general appearance. From day four PI and thereafter, all mice appeared normal at all observation time points. In summary, two female mice died. Three male and two female mice were found moribund and were euthanized.

Non-human Primate Toxicology

Ten (10) cynomolgus monkeys (*Macaca fascicularis*) were used in Protocol No. 10-01-10681, titled "GLP Study Prior to Phase I Clinical Trials for DOTAP: Cholesterol-*Fus 1* Liposome Complex (DOTAP: Chol/*Fus 1*) Relevant Safety Study". Six experimental animals (three male and three female) were injected with DOTAP:Chol/*Fus 1* complex on Day 1 and Day 21 of the study. Four control animals (two male and two female) were injected with DOTAP:Cholesterol alone on Day 1 and Day 21 of the study. At days 46-52 the animals were necropsied, blood was collected for hematology and chemistries, and organs were collected for histopathological analysis.

Significant gross and microscopic lesions were found in 1/10 monkeys on protocol. This animal received 1 dose of 0.6 mg/kg DNA, DOTAP: Chol (high dose) and died within 18-20 hours. Lesions in this monkey were most likely treatment related. A second monkey that received the high dose of DNA, DOTAP: Chol had changes in a lymph node. The significance of these minimal changes is not known. Equivocal lesions were found in the femoral bone marrow of two low dose (0.2 mg/kg DNA, DOTAP: Chol) monkeys. The latter may be incidental findings, but were not seen in other protocol animals. No significant gross or microscopic lesions were found in the remaining six animals that received either DOTAP: Chol only or 0.2 mg/kg DNA, DOTAP: Chol.

Anticipated Human Toxicity

The results of the above preclinical toxicity data suggest that possible human organ toxicities may involve lymphoid tissue and spleen (necrosis, apoptosis, and atrophy) and liver (acute necrosis). Kidney toxicity (acute tubular necrosis, glomerulonephritis) was less frequently observed in mice. Of note, the finding of multifocal myocardial degeneration, necrosis, and mineralization observed in the mice were judged to be most likely incidental findings by the veterinary pathologist, since this has been found to occur in control C3H mice.
Clearly, trying to predict human toxicity remains speculative. The extremely rapid infusion rates used in mice remains a potential confounding factor when interpreting the preclinical toxicity data. The starting dose level for this phase I study will be the equivalent of 1/10 of the LD_0 in monkeys.

3.1 Drug Formulation

DOTAP:Chol-*fus1* complex will be produced under GMP like conditions in the UTMDACC Blood & Marrow Transplantation GMP facility, Houston, TX under the supervision of Dr. John D. McMannis (713-563-4800). In addition to safety testing, preparations will be tested for particle size, DNA content, and in vitro efficacy.

DOTAP:Chol-*fus1* complex will be provided as a liquid form. A maximum of 150 ug DNA will be encapsulated in 4mM DOTAP:Chol complex. Prior to mixing, the individual products (DNA and liposome) are stored at + 4oC. Following mixing, the products are stored at room temperature and used within 24 hours. The drug is prepared in clinical grade 5% dextrose.

DOTAP:Chol-fus 1 will be mixed with 10% overfill for a total volume of 110cc. 5cc of the overfill will be removed and archived in the UTMDACC Blood & Marrow Transplantation GMP facility for Dr. Jack Roth's research lab at UTMDACC. 5cc of the overfill will be removed for sterility testing and gram stain at the MDACC Microbiology Laboratory. The overfill will be transported from the UTMDACC Blood & Marrow Transplantation GMP facility to the MDACC Microbiology Laboratory using the GMP facilities' SOP for transport. Final dose will not be released from UTMDACC Blood & Marrow Transplantation GMP facility unless results of STAT gram stain are reported as negative, defined as no visible microorganisms,. A faxed or e-mailed hard copy preliminary report of the gram stain will be provided to the UTMDACC Blood & Marrow Transplantation GMP facility before the GMP facility releases the drug. Any doses with a positive gram stain result will be disposed of in a red biohazard box. The drug will be transported from the UTMDACC Blood & Marrow Transplantation GMP facility to the CTRC for patient infusion using the GMP facilities' SOP for transport.

4.0. Patient eligibility

4.1. Inclusion Criteria

To be eligible for participation in the study, patients must meet all of the following criteria:

1. Histologically or cytologically documented non-small cell lung cancer (NSCLC) or small cell lung cancer (SCLC)

2. **For NSCLC subjects:** Locally advanced, unresectable, incurable stage IIIB (pleural effusion) or stage IV NSCLC, or recurrent NSCLC that is not potentially curable by radiotherapy or surgery. Patients must have received at least one prior platinum-based chemotherapy regimen for NSCLC. **For SCLC subjects:** Extensive disease or recurrent disease after initial treatment for limited disease. Patients must have received prior platinum-based chemotherapy or chemoradiotherapy. **All subjects:** There is no limit to the number of prior chemotherapy regimens received.

3. Preference will be given to patients with tumors amenable to biopsy. In the expansion cohort at MTD, all patients must have tumor amenable to biopsy and must consent to biopsy.

4. Karnofsky Performance Status ≥ 70%, or Zubrod Performance status ≤ 1 (see Appendix A).

5. Patients must have voluntarily signed an informed consent in accordance with institutional policies.

6. Negative serum pregnancy test (serum HCG) if female and of childbearing potential (non-childbearing is defined as greater than one year post-menopausal or surgically sterilized). Since beta-HCG may be falsely elevated as a result of malignancy, women of child-bearing potential who have an elevated serum beta-HCG level are eligible for enrollment if they have two Transvaginal Ultrasound (TVUS) scans one week apart along with serial beta-HCG levels two weeks apart that are inconsistent with pregnancy and a Gynecology consult to ensure that the beta- HCG level was at a value high enough to see pregnancy with TVUS. Subjects are required to agree to practice effective birth control during the study period.

7. Negative serology for Human Immunodeficiency Virus.

8. Patients must be ≥ 4 weeks beyond major surgical procedures such as thoracotomy, laparotomy or joint replacement, and must be ≥ 1.5 weeks beyond minor surgical procedures such as biopsy of subcutaneous tumors, pleuroscopy, etc, and must not have evidence of wound dehiscence, active wound infection, or comparable major residual complications of the surgery.

9. ANC > 1500 x $10^9/mm^3$, plt count > 100,000 x $10^9/mm^3$

10. PT and PTT < 1.25 times the institutional upper limit of normal.

11. Adequate renal function documented by serum creatinine of ≤ 1.5 mg/dl or calculated creatinine clearance > 50 ml/min

12. Adequate hepatic function as documented by serum bilirubin< 1.5 mg/dl and SGOT and SGPT ≤ 1.5 X upper limit of normal.

13. Patients with asymptomatic brain metastases that have been treated are eligible if the following criteria are met: No history of seizures in the preceding 6 months. Definitive

treatment must have been completed ≥4weeks prior to registration. Subjects must be off steroids that were being administered because of brain metastases or related symptoms for ≥2 weeks. Post-treatment imaging within 2 weeks of registration must demonstrate stability or regression of the brain metastases.

14. Stable cardiac condition with a left ventricular ejection fraction > 50%.

15. FEV1 and corrected DLCO of ≥ 40% of predicted.

4.2. Exclusion Criteria

Patients with any of the following will not be eligible for enrollment:

1. Females who are pregnant or breast-feeding.

2. Patients who received investigational therapy (agents that are not FDA approved), monoclonal antibody such as bevacizumab or cetuximab, or who received radiotherapy to the skull, spine, thorax or pelvis within 30 days of entry into the protocol. Patients are permitted to have received palliative radiotherapy to an extremity provided at least 14 days has elapsed since completion of therapy, provided the patient received no more than 10 radiotherapy fractions and a dose no higher than 30 Gy to that site, and provided skull, spine, thorax or pelvis were not in the radiotherapy field.

3. Patients who have received standard chemotherapy with FDA approved agents within 21 days of entry into the protocol.

4. Patients who have received therapy with an oral tyrosine kinase inhibitor (eg, erlotinib) within 14 days prior to entry into the protocol.

5. Active systemic viral, bacterial or fungal infections requiring treatment.

6. Patients with brain metastases (except as allowed in section 4.1.13). Neurological assessment will be used to determine brain metastases.

7. Patients with serious concurrent illness or psychological, familial, sociological, geographical, or other concomitant conditions that , in the opinion of the investigator, would not permit adequate follow-up and compliance with the study protocol.

8. Use of any investigational agent within four weeks of study treatment.

9. Prior gene therapy.

10. History of myocardial infarction within 6 months, angina within the past 6 months, or a history of arrhythmias on active therapy.

5.0. Treatment Plan

5.1 A starting dose for patients of 0.02mg/Kg given intravenously was selected based on extrapolations from the non-toxic non-human primate study to humans (1/10 LD_0). Beginning 12/2003, the starting dose has been amended to 0.01 mg/Kg. The rationale for this change is outlined in the FDA clinical hold letter dated October 6, 2003. This clinical trial will be a dose escalation trial. The trial design will be based on a continuous reassessment model (CRM) which allows the maximum tolerated dose to be periodically re-estimated (see Section 10). Patients entered at a given dose level will not be eligible for dose escalation while on study. A cohort of 3 patients will be treated at each dose level. After treating 3 patients at a given dose level, the patients will be observed for 2 weeks to evaluate the toxicity. The information of whether the patients develop dose limiting toxicity (DLT) will be recorded for computing the posterior probability of toxicity given the prior and the data. Therefore only toxicity during cycle #1 will be used to determine the next dose level. The next cohort of patients will be treated at the dose level having the posterior probability of DLT closest to the pre-specified target toxicity level (TTL). All the patients will be treated in a dose-escalation fashion starting from the lowest level. The next dose level can be moved up if indicated by the calculation of the posterior distribution but no skipping of doses is allowed. A maximum of 51 patients will be treated in this Phase I trial. The dose for subsequent trials will be the MTD.

All doses will be diluted in 100ml D5W and will be infused in a peripheral vein over 25-35 minutes. The patients will be treated in an outpatient infusion area. The patient's pulse, blood pressure, and general condition will be monitored before infusion, at 15 minutes into infusion, and after the infusion. Monitoring will be under the supervision of an attending physician.

Patients may receive up to six treatments at their specified dose level. The time interval between treatments is 3 weeks +/- 2 days, with administration of treatment occurring on day 1. Treatment will be stopped for any cumulative grade 3 (grade 4 hematologic) or greater toxicities that meet the definition of dose-limiting toxicity, as outlined in section 10.2, or for disease progression. Evaluation and measurement of primary and other disease sites with appropriate examination (clinical or radiological including CT scans) will be done every two treatment cycles (6 weeks). Patients will receive treatment as outpatients and will be monitored for two to six hours after each injection.

Patients that continue to demonstrate a symptomatic or biologic response to treatment at the completion of six courses may be continued on treatment at the discretion of the treating physician and the principle investigator in collaboration with advisors at the FDA. In each instance these patients will be considered on a case by case basis.

Premedications: The protocol was reopened to accrual in April 2004 after being placed on a clinical hold by the FDA. After reopening the protocol, the first cohort of three patients were treated at a reduced starting dose level of 0.01 mg/Kg without the use of premedications. Two of three subjects developed evidence of an inflammatory response characterized by fever (grade 3), chills, and hypotension (grades 2 and 3). Prior to the clinical hold, a total of six subjects had been treated with a total of 15 treatment cycles.

One subject developed a grade 2 fever with the first cycle of treatment. Dexamethasone and diphenhydramine premedications were subsequently used for the remaining 14 treatment cycles, and the highest observed fever was grade 1, which occurred in 3 subjects (4 cycles).

The protocol was subsequently amended to require that all subjects begin the following medication regimen before DOTAP:Chol-*fus1* treatment, starting at the same initial dose level of 0.01 mg/Kg:

Dexamethasone 8mg PO 24 and 12 hours before treatment and 12, 24 and 36 hours after treatment (total number doses = 5).
Dexamethasone 20mg IV 30 minutes prior to treatment
Diphenhydramine 50mg PO/IV 30 minutes prior to treatment

5.2 Biopsies:

5.2.1 Optional Biopsies During Dose Escalation Phase: Pre- and 24 hour post-treatment biopsies of accessible tumor (and normal bronchial mucosa, if biopsy is obtained by bronchoscopy) will be obtained for fixation and snap freezing in liquid nitrogen for analysis of 3p LOH and quantitative PCR and reverse-transcriptase PCR using the PE Biosystems Prism 7700. For participants that agree to the optional biopsies an additional 30cc of blood will be drawn in Vacutainer CPT tubes for isolation of peripheral blood mononuclear cells for comparison analysis.

5.2.2 Biopsies During Dose Expansion Cohort: Once MTD is defined, 6 additional consenting patients tumor amenable to biopsy and who consent to biopsy will be entered. Pre- and 24 hour (+/- 4 hours) post treatment biopsies of accessible tumor (and normal bronchial mucosa, if biopsy is obtained by bronchoscopy) will be obtained for fixation and snap freezing in liquid nitrogen for analysis of 3p LOH and quantitative PCR and reverse-transcriptase PCR using the PE Biosystems Prism 7700. An additional 30cc of blood will be drawn in Vacutainer CPT tubes for isolation of peripheral blood mononuclear cells for comparison analysis.

5.3 DOTAP:Chol-*fus1* liposomes will be produced under GMP like conditions in the UTMDACC Blood & Marrow Transplantion GMP facility, under the supervision of Dr. John D. McMannis (713-563-4800). In addition to safety testing, preparations will be tested for particle size, DNA content, and in vitro efficacy.

5.4 Management plan infusion/allergic reactions to DOTAP:chol-Fus1.
Prior to administering DOTAP:Chol-*Fus1* the following medications will be at the bedside for possible allergic reactions: Epinephrine (1:1000) 0.5 ml IV, diphenhydramine 50 mg IV, and hydrocortisone 100 mg IV.

Allergic/Hypersensitivity reactions will be managed as outlined in Table 1 below.

Table 1. Management of Allergic/Hypersensitivity Reactions

Severity of Symptoms	Treatment Guidelines
Mild Symptoms:<br>Localized cutaneous reaction, mild pruritus, mild flushing, mild rash, fever ≤38ºC	Interrupt infusion, alert attending physician<br>Give diphenhydramine 50 mg IV, dexamethasone 10 mg IV, cimetidine 300 mg IV. Acetominophen 650 mg PO if needed.<br>Resume infusion at 50% reduced rate, monitor for worsening of symptoms<br>Give future infusions with the following premedications 30 min prior: diphenhydramine 50 mg IV, dexamethasone 20 mg IV, cimetidine 300 mg IV.<br>Begin future infusions at 50% reduced rate, may increase rate every 15 minutes (in 25% increments) if no hypersensitivity reactions
Moderate Symptoms:<br>Any symptom not listed above (mild symptoms) or below (severe symptoms) such as generalized pruritus, flushing, rash dyspnea, hypotension with systolic BP > 80 mm Hg, fever > 38ºC	Interrupt infusion, alert attending physician<br>Give diphenhydramine 50 mg IV, dexamethasone 10 mg IV, cimetidine 300 mg IV. IV fluids, Acetominophen 650 mg PO if needed.<br><br>Monitor patient until resolution of symptoms<br>If physician assesses patient to be stable, may resume infusion at 75% reduced rate, monitor for worsening of symptoms<br>Give future infusions with the following premedications 30 min prior: diphenhydramine 50 mg IV, dexamethasone 20 mg IV, cimetidine 300 mg IV.<br>Begin future infusions at 75% reduced rate, may increase rate every 15 minutes (in 25% increments) if no hypersensitivity reactions
Severe Symptoms:<br>Bronchospasm, generalized urticaria, hypotension with systolic BP< 80 mm Hg, angioedema, anaphylaxis	Discontinue infusion, alert attending physician<br>Give diphenhydramine 50 mg IV, dexamethasone 10 mg IV, cimetidine 300 mg IV. Give epinephrine 0.5 mg IV/SC, IV fluids if needed.<br>Transfer to Emergency Unit or Intensive Care Unit if deemed appropriate by attending physician.<br>Monitor patient until resolution of symptoms<br>No further therapy on this trial.

For other infusion toxicity reactions not included in the above table, the following guidelines will be employed as outlined in Table 2 below.

Table 2. Management of Other Infusion Reactions

Infusion Toxicity Grade	Treatment Guidelines

Grade 1	Alert attending physician.<br>Treat any symptoms as clinically indicated per attending physician<br>May continue infusion at 50% reduced rate if approved by attending physician.
Grade 2	Alert attending physician.<br>Treat any symptoms as clinically indicated per attending physician<br>If toxicity improves to $\leq$ grade 1, may resume infusion at 75% reduced rate if approved by attending physician.
$\geq$ Grade 3	Alert attending physician.<br>Treat any symptoms as clinically indicated per attending physician<br>No further therapy on this trial.

5.5 Patients must fulfill the entry eligibility criteria as listed in section 7.0 in order to be eligible for repeated treatment. Patients with toxicities that are judged by the investigator to be probably or definitely due to the study agent and that have not recovered to baseline or grade 0-1 will have treatment delayed for one week. Patients with toxicities that are judged by the investigator to be possibly due to the study agent and that have not recovered to baseline or grade 0-1 will also have treatment delayed for one week unless the adverse event or toxicity is judged by the PI to be more likely due to the patient’s underlying medical conditions or other concurrent medications. Patients who have treatment withheld due to toxicity for more than two weeks will be removed from the study. If a patient’s treatment is withheld for any reason other than toxicity, progression, or study completion, (eg. drug not available, patient had to reschedule trip to Houston) treatment may be restarted at a later time at the discretion of the PI and the treating physician.

5.6 Patients who experience dose-limiting toxicity will not receive any further treatment unless they exhibit radiographic evidence of reduction in tumor size, without any documented areas of tumor growth. For patients with dose-limiting toxicity, but with radiographic evidence of tumor shrinkage (and with no documented areas of tumor growth), retreatment will be permissible at 50% of the dose giving rise to the dose-limiting toxicity.

5.7 As noted above, once MTD is determined (as defined in section 10.4), 6 additional patients will be entered in an expansion cohort at MTD to confirm safety and to further assess biological correlates. To be entered on the expansion cohort, patients must have tumor amenable to biopsy before and after treatment and must consent to these biopsies.

6.0. Pretreatment Evaluation

6.1. Enrollment

All patients who are screened for study enrollment and who have signed an informed consent will be recorded on a Screening Log maintained in the Investigator Study File. Patients who do not meet all eligibility criteria for study enrollment or who withdraw consent prior to receiving

study treatment will be considered screen failures. Patients who meet all eligibility criteria and have signed an informed consent will be entered into the study.

6.2. Screen/Baseline Evaluation

The screen evaluation includes procedures that are necessary to determine patient eligibility for study treatment.

The baseline evaluation (baseline) is defined as an assessment of patient status prior to any study treatment. Results obtained during the screen evaluation may also be used for the baseline evaluation on cycle 1.

The following table (Table 3) outlines all procedures that will be performed for the screening and baseline evaluations. All of the listed procedures must be conducted within the specified time frames and results obtained prior to study treatment:

Table 3: Screening/Baseline Evaluations

**Procedures**		**Timing**
Informed Consent	Obtain patient informed consent before any study specific procedures are performed.	Prior to any study specific procedures.
Medical History	Including: Diagnosis of primary disease, diagnosis of malignant lesions, concurrent illness, prior anticancer treatment, weight loss within last 6 months	Within 30 days of study treatment
Physical Examination	Including: height, weight, Karnofsky or Zubrod performance status, vital signs (temperature/pulse/sitting blood pressure/respiration rate)	Within 14 days of study treatment
Concomitant Medications	Document all medications the patient reports having taken during the 30 days prior to study treatment, including OTC and PRNs	At time of patient enrollment
Signs & Symptoms	Document all clinical signs and symptoms the patient reports as having been present during the 30 day period prior to study treatment	At time of patient enrollment
Hematology	CBC with differential, platelets, prothrombin time, partial thromboplastin time	Within 14 days of study treatment
Biochemistry	Calcium, sodium, potassium, chloride, phosphate, total protein, albumin, creatinine, alkaline phosphatase, SGOT (AST), SGPT (ALT), lactate dehydrogenase (LDH), urea, total bilirubin, phosphorus, and magnesium	Within 14 days of study treatment
Serum HCG	If female of child-bearing potential	Within 7 days of study treatment
Cardiac Assessment	EKG and MUGA scan or Echocardiogram	Within 21 days of study treatment
HIV Serology	ELISA for HIV 1 (Western Blot if positive)	Within 21 days of study treatment
Urine	Routine Urinalysis	Within 14 days of study treatment
Respiratory Assessment	FEV1, corrected DLCO, and pulse oximetry (oxygen saturation)	Within 14 days of study treatment
Tumor Assessment[1]	Chest x-ray<br>Chest CT scan (or PET/CT)<br>Other radiologic studies (abdominal or pelvic CT, brain CT or MRI, bone scan) as appropriate based on other sites of known or clinically suspected disease.	Within 21 days of study treatment<br>Within 21 days of study treatment<br>Within 21 days of study treatment
Biopsies (not required, although preference will be given to patients with tumors amenable to biopsy) An additional 30 cc blood sample is drawn on patients that elect to have the biopsy.	Histology and immunohistochemistry<br>Biopsy (core, incisional, excisional, or forceps) of accessible tumor and adjacent normal mucosa for endobronchial lesions for vector specific RT-PCR, and western blots.	Can be from original diagnostic biopsy<br>Within 21 days prior to study treatment<br><br>Blood: Within 21 days of study treatment on the same day as the biopsy whenever possible.

[1] To ensure comparability, radiologic studies to assess response should be performed using comparable techniques to the extent that is practical

7.0. Evaluation During Study

On-study evaluations include all procedures performed after the patient has been enrolled until patient is off-study (see definition section 7.3). Patients will be evaluated for toxicity by history, physical examination, and laboratory parameters (CBC with differential and platelets, serum chemistry, urine analysis and EKG) prior to each treatment. Results must be evaluated and the results must continue to meet eligibility criteria prior to retreatment. (Note: To undergo retreatment, patients will not require repeat serum pregnancy test, serology for HIV, assessment of left ventricular ejection fraction, FEV1 and corrected DLCO, unless clinically indicated in the opinion of the investigator.)

UTMDACC nurses will assess the patient and document vital signs and any adverse events on post treatment days 2, 3 and 8 (+/- 1 day). In addition, a CBC with differential and platelets, and a serum chemistry will be done on days 2, 3 and 8 (+/- 1 day).

7.1. The following table (Table 4) gives an overview of the procedures that will be conducted at specified time intervals during the study:

Table 4: Evaluations during Treatment Period

Procedures		Timing
History and Physical Exam	Physical Examination including: weight, Karnofsky or Zubrod performance status	Prior to each treatment
Concomitant Medications	Document any changes in concomitant therapy	Prior to each treatment
Adverse Events	Document any clinically significant signs or symptoms which develop or worsen from baseline. Vital signs.	Prior to each treatment and on days 2, 3, and 8 (+/- 1 day) following treatment.
Hematology	CBC with differential, platelets,	Prior to each treatment and on days 2, 3, and 8 (+/- 1 day) following treatment.
Biochemistry	Calcium, sodium, potassium, chloride, phosphate, total protein, albumin, creatinine, alkaline phosphatase, SGOT, SGPT, lactate dehydrogenase (LDH), urea, total bilirubin, phosphorus, and magnesium	Prior to each treatment and on days 2, 3, and 8 (+/- 1 day) following treatment.
Respiratory Assessment	Pulse oximetry (oxygen saturation)	Prior to each treatment
Urine	Routine urinalysis	Prior to each treatment.
Cardiac Assessment	EKG	Prior to each treatment.
Tumor Assessment	Chest x-ray<br>Chest CT scan (or PET/CT)<br><br>Other radiologic studies (abdominal or pelvic CT, brain CT or MRI, bone scan) as appropriate based on other sites of known or clinically suspected disease.<br><br>Bone scan	Prior to each treatment<br>After every second treatment cycle<br>After every second treatment cycle<br><br>Will not be used to evaluate response, but may be repeated as

		clinically appropriate to help document clinically suspected new or worsening bone lesions
Biopsies (not required during the initial dose-escalation phase of the study, although preference will be given to patients with tumors amenable to biopsy; required during the dose expansion cohort at MTD)	Extraction DNA/RNA/protein: biopsy (core, incisional, excisional or forceps) of accessible tumor (and of bronchial and adjacent normal mucosa for endobronchial lesions) for vector specific RT-PCR and western blots<br><br>Biopsy **(core, incisional, excisional or forceps)** for histology and TUNEL staining and immunohistochemistry	24 hrs (+/- 4 hours) following treatment for accessible tumor after the first injection only<br><br>24 hrs (+/- 4 hours) following treatment for accessible tumor after the first injection only.
An additional 30 cc blood sample is drawn on patients that elect to have the biopsy.	Lymphocytes will be separated and used for PCR and gene transduction to determine their ability to take up the vector and express their transgene.	Blood is drawn the same day as the biopsy.

7.2 Discontinuation of Study Treatment

The following are reasons for discontinuing study treatment:

· Consent withdrawn.
· The patient experiences dose-limiting toxicity, as defined in 10.2. If a DLT is reversible in one week or less, is not life threatening, is not due to the patient experiencing a hypersensitivity reaction and there is evidence of therapeutic benefit, a patient may continue on treatment at a 50% dose reduction.
· Significant hemoptysis (requiring inpatient observation and treatment).
· Coagulopathy (defined for the purposes of this study as PT or PTT > 1.25 times the upper limit of normal, hemorrhage requiring transfusion or hospital admission, or venous or arterial thromboembolic events requiring therapy).
· Progressive disease.
· Progressive postobstructive pneumonia requiring initiation of intravenous antibiotics.
· Investigator decision.
· Non-compliance by patient with protocol requirements.
· Administration of any cytotoxic therapeutic agent or experimental drug during the study.
· Pregnancy.

The reason for discontinuation of study treatment will be documented for each patient. Patients who withdraw from the study prematurely will not be replaced if they have received at least one dose of study drug and have developed dose-limiting toxicity or are evaluable for toxicity for cycle 1 of therapy. If a DLT is reversible in one week or less, is not life threatening, is not due to the patient experiencing a hypersensitivity reaction and there is evidence of therapeutic benefit, a patient may continue on treatment at a 50% dose reduction.Patients who withdraw from the study prematurely and who have not received study drug or who have not developed dose-limiting toxicity and who are not evaluable for toxicity for cycle 1 of therapy may be replaced if deemed appropriate by the investigator.

Patients completing the entire 6 cycles of treatment will be assessed on days 2, 3, 8, 15 and 31 following treatment. Day 8 will have a +/-1 day window and days 15 and 31 will have a +/-3 day window. Patients who discontinue treatment >/=15 days into any given cycle will be assessed on that day and again at 31 days (+/- 3 days) following their final dose of DOTAP:Chol-Fus 1 therapy. The day 31 visit is to review toxicities and to determine if the patient has started on another treatment regimen, this can be done by phone if coming to the clinic creates a hardship for the patient. Patients who go off-study will be provided with a calendar with follow-up appointments clearly marked. The importance of these visits will be stressed to subjects by the research staff and reasons for lack of compliance (eg, patient refusal or patient inability to travel) will be documented. Patients who discontinue due to one or more adverse event(s) (AE) will be followed until resolution or stabilization of the event.

7.3. Off-Study Definition

Patients who have study treatment discontinued or who have completed three months of observation will be considered off-study.

7.4. Post-Study Follow-Up Evaluations

Once off-study, patients will be contacted by phone at three month intervals, until patient death or patient is lost to follow up, to obtain vital status and interval cancer treatment history.

8.0. **Adverse Events**

8.1. Definitions

Adverse Event: Any symptom, sign, illness, or experience which develops or worsens in severity and/or frequency during the course of the study (i.e., any change from baseline). Intercurrent illnesses or injuries should be regarded as adverse events. Abnormal results of diagnostic procedures are considered to be adverse events if the abnormality results in study withdrawal, is associated with clinical signs or symptoms, leads to treatment or to further diagnostic tests, or is considered by the investigator to be of clinical significance.

Expected Adverse Events: Adverse events that are known to occur as a result of the study agent or planned premedications

The following are Expected Adverse Events for the study agent alone, based on observations in earlier patient cohorts:

- Fever and chills
- Hypotension

The following are Expected Adverse Events for the Dexamethasone given intravenously and orally before and/or after the study agent:

- Hyperglycemia
- Lymphopenia, reduced eosinophil and basophil counts

- Elevated neutrophil counts
- Hypokalemia
- Nausea, vomiting, dyspepsia, gastric irritation, peptic ulcer
- Mood and sleep disturbances or alterations (rarely psychosis)
- Appetite stimulation
- Hypertension, edema
- Acneiform rash
- Flushing or skin tingling with intravenous administration

The following are Expected Adverse Events for the Diphenhydramine given intravenously or orally before the study agent:

- Sedation, dizziness, incoordination, confusion, blurred vision, tremor
- Palpitations, tachycardia

Unexpected Adverse Event: Any adverse event that is not identified in nature, severity, or frequency in the current protocol.

Serious Adverse Event: Any adverse drug experience occurring at any dose that results in any of the following outcomes:

- Death
- A life-threatening adverse drug experience – any adverse experience that places the patient, in the view of the initial reporter, at immediate risk of death from the adverse experience as it occurred. It does not include an adverse experience that, had it occurred in a more severe form, might have caused death.
- Inpatient hospitalization or prolongation of existing hospitalization
- A persistent or significant disability/incapacity – a substantial disruption of a person's ability to conduct normal life functions.
- A congenital anomaly/birth defect.

Important medical events that may not result in death, be life-threatening, or require hospitalization may be considered a serious adverse drug experience when, based upon appropriate medical judgment, they may jeopardize the patient or subject and may require medical or surgical intervention to prevent one of the outcomes listed in this definition. Examples of such medical events include allergic bronchospasm requiring intensive treatment in an emergency room or at home, blood dyscrasias or convulsions that do not result in inpatient hospitalization, or the development of drug dependency or drug abuse (21 CFR 312.32).

- Important medical events as defined above, may also be considered serious adverse events. Any important medical event can and should be reported as an SAE if deemed appropriate by the Principal Investigator or Sponsor.

- The departmental research team responsible for the conduct of this protocol will immediately notify City of Hope within 24 hours after it becomes aware of any serious

unanticipated adverse clinical event resulting from either the use of Plasmid DNA Materials or the failure of the study team, institution, or entity to comply with any appropriate statute, requirement, or professional standard relating to the use of the Plasmid DNA Materials. All efforts to de-identify the patient's information must be used to the extent that is possible.

8.2. Eliciting Adverse Event Information

Adverse events will be elicited at each clinic visit during participation in the study. All adverse events that are directly observed and all adverse events that are spontaneously reported by the patient are to be documented by the investigator.

8.3. Grading/Rating Scale

All adverse events reported during the study will be evaluated and graded on a scale of 1-4. The graded toxicity scale used in this study is the CTC version 2.0 for toxicity and Adverse Event reporting. A copy of the CTC version 2.0 can be downloaded from the CTEP home page (http://ctep.info.nih.gov).
For adverse events not covered by the CTC, the following definitions will be used:

Grade	Rating	Description
1	Mild	Adverse event is transient and easily tolerated by the patient; asymptomatic
2	Moderate	Adverse event causes the patient discomfort and interrupts the patients usual activities; symptomatic but does not interfere with function
3	Severe	Adverse event causes considerable interference with the patients usual activities
4	Life-threatening	Adverse event is incapacitating or life-threatening

8.4. Reporting of Adverse Events

Pre-existing conditions will be recorded on Pre-Treatment Signs and Symptoms case report form. All adverse events, regardless of severity, that occur during the course of the study will be

recorded on the Adverse Event case report forms. In order to avoid vague, ambiguous or colloquial phrases, the event should be recorded using standard medical terminology rather than the patient's own words. The investigator's assessment of the relationship of an adverse event to study treatment does not affect the definition of an adverse event.

8.5. Serious Adverse Event Reporting (SAE)

A serious adverse event is – any adverse drug experience occurring at any dose that results in any of the following outcomes:

- Death
- A life-threatening adverse drug experience – any adverse experience that places the patient, in the view of the initial reporter, at immediate risk of death from the adverse experience as it occurred. It does not include an adverse experience that, had it occurred in a more severe form, might have caused death.
- Inpatient hospitalization or prolongation of existing hospitalization
- A persistent or significant disability/incapacity – a substantial disruption of a person's ability to conduct normal life functions.
- A congenital anomaly/birth defect.

Important medical events that may not result in death, be life-threatening, or require hospitalization may be considered a serious adverse drug experience when, based upon appropriate medical judgment, they may jeopardize the patient or subject and may require medical or surgical intervention to prevent one of the outcomes listed in this definition. Examples of such medical events include allergic bronchospasm requiring intensive treatment in an emergency room or at home, blood dyscrasias or convulsions that do not result in inpatient hospitalization, or the development of drug dependency or drug abuse (21 CFR 312.32).

- **Important medical events as defined above, may also be considered serious adverse events. Any important medical event can and should be reported as an SAE if deemed appropriate by the Principal Investigator or the IND Sponsor, Office of Research Education and Regulatory Management (ORERM).**
- All events occurring during the conduct of a protocol and meeting the definition of a SAE must be reported to the IRB in accordance with the timeframes and procedures outlined in "University of Texas M. D. Anderson Cancer Center Institutional Review Board Policy on Reporting Serious Adverse Events". Unless stated otherwise in the protocol, all SAEs, expected or unexpected, must be reported to ORERM, regardless of attribution (within 5 working days of knowledge of the event).
- **All life-threatening or fatal events,** expected or unexpected, and regardless of attribution to the study drug, must have a written report submitted within **24 hours** (next working day) of knowledge of the event to the Safety Project Manager in ORERM.
- **The MDACC "Internal SAE Report Form for Prompt Reporting" will be used for reporting to ORERM.**

- **Serious adverse events will be captured from the time the patient signs consent until 30 days after the last dose of drug. Serious adverse events must be followed until clinical recovery is complete and laboratory test have returned to baseline, progression of the event has stabilized, or there has been acceptable resolution of the event.**
- **Additionally, any serious adverse events that occur after the 30 day time period that are related to the study treatment must be reported to ORERM. This may include the development of a secondary malignancy.**
- **The gene therapy reporting addendum ("Additional Reporting Form for Serious Adverse Events on Gene Transfer Trials") must be included with each SAE submitted.**

Reporting to FDA:

- Serious adverse events will be forwarded to FDA by the IND Sponsor (Safety Project Manager ORERM) according to 21 CFR 312.32.

It is the responsibility of the PI and the research team to ensure serious adverse events are reported according to the Code of Federal Regulations, Good Clinical Practices, the protocol guidelines, the sponsor's guidelines, and Institutional Review Board policy.

One of the following individuals should be contacted if a patient on this trial is known or suspected to have experienced an adverse event:
Mary Ann Gianan, RN, phone – 713-563-9152
Karen Smith, RN, phone- 713-563-9156
Rhodette Francisco, R.N., C.C.R.P.; phone – 713-563-9155
David J. Stewart, M.D. 713-792-6363
Jack A. Roth, M.D.; phone – 713-792-7664
Fax - 713-792-4188 (fax)

At the time of the initial report the following information should be provided if possible: protocol number, study site, patient number, study phase during which the event occurred, description of the event, date of onset and current status, start date of treatment, whether treatment has been discontinued, reason why the event is classified as serious, and the investigator's current assessment of the relationship between the event and study treatment.

8.6. In Case of Death

Where feasible and appropriate, an autopsy will be requested on patients who die while on study. DNA will be extracted from tumor and normal tissues to determine if vector-specific genes are present and expressed. PCR amplification of specific sequences will be used to determine this. A biopsy of the indicator lesion will be performed for histology.

9.0. Criteria for Response

9.1.1. Definitions of Measurable Disease

Measurable disease is defined as tumor masses with identifiable diameters measurable in two dimensions by computed tomography. All sites of disease (up to a maximum of five lesions per organ and 10 lesions in total, designated the "target lesions") must be followed and recorded in the source documents and on the patients case report form. Measurable lesions must be 1 cm in at least one dimension.

All tumor measurements must be recorded in millimeters. The recorded measurement will be the longest diameter and its perpendicular applied at the widest portion of the tumor. Size will be reported as the product of the diameters.

9.1.2. Response Classifications

All target lesions must be reported and assessed. Any tumor response assessment should consider all available target lesion assessments. Final assessments identical to those performed before study entry will be performed one month after completion of treatment.

Complete Response (CR)
Complete disappearance of all clinical evidence of lesion (both target and non-target) by CT, MRI, or PET scans for a minimum of four weeks. This means that an intermediate visit with appropriate assessments should be planned in advance once the CR has been declared. At the time of confirmation, all sites of disease must be re-evaluated.

Partial Response (PR)
A fifty percent or greater decrease in the sum of the products of the perpendicular diameters of measurable target lesions. This means that an intermediate visit with appropriate assessments should be planned in advance once the PR has been declared. At the time of confirmation, all target lesions must be re-evaluated.

Progressive Disease (PD) / Treatment Failure
An increase of 25% in the sum of the products of the diameters of the measured target lesions or appearance of new lesions.

Stable Disease (SD) / No Change
Stable disease is defined as any variation of the indicator lesion not meeting the criteria of a complete or partial response or progressive disease.

9.1.3. Determination of Overall Response

Best overall response is the best response designation recorded from the start of treatment until disease progression. Complete and partial responses have to be confirmed by two evaluations of the disease taken at least four weeks apart.

9.1.4. Measures of Duration

Response Interval
The response interval is measured from the date when the response is first noted until the last date at which the measurements satisfy the requirements for a partial or complete response or stable disease.

Ongoing Response
An ongoing response is defined as a responder for whom no end of response can be determined. In this case the duration of response is considered as the last date when there is no evidence of a continuing response.

Time to Progression
The time to progression will be measured from the date of first study treatment to the documentation of progressive disease.

Overall Survival
The duration of overall survival will be determined by measuring the time interval from the date of first treatment to date of death, irrespective of the cause of death.

10.0. **Statistical Consideration:**

The primary objective for this Phase I study is to evaluate the toxicity of the Intravenous DOTAP:Chol-*fus1* complex and to determine the maximum tolerated dose (MTD) for the subsequent Phase II trials. We will apply the continual reassessment method (CRM) for this trial. (O'Quigley et al., 1990) The key elements of the design are described below.

10.1. Starting Dose

For additional safety considerations, the starting dose is set at 1/10 of the non-human primate LD_0. The starting dose is at 0.02 mg/Kg. Beginning 12/2003, the starting dose has been amended to 0.01 mg/Kg. The rationale for this change is outlined in the FDA clinical hold letter dated October 6, 2003.

10.2. Toxicities

The NCI Common Toxicity Criteria 2.0 will be used. The dose limiting toxicity (DLT) is defined as grade 3 or greater non-hematological or hematological toxicity. Toxicity that is attributed to the dexamethasone or diphenhydramine premedication will **not** be considered DLT. Grade 3 fatigue or weakness lasting < 4 days will also **not** be considered DLT even if attributed to the study drug. Asymptomatic hypophosphatemia, regardless of grade will not be considered a DLT if it can be corrected to grade 0 – 1 in 4 days or less. Also, toxicities or adverse events that are judged by the PI to be more likely due to the patient's underlying medical conditions or other concurrent medications will not be judged to be DLTs. If a DLT is reversible in one week or less, is not life threatening, is not due to the patient experiencing a hypersensitivity reaction and there is evidence of therapeutic benefit, a patient may continue on treatment at a 50% dose reduction.

10.3. Dose-toxicity curve

We choose the exponential power family to model the dose toxicity relationship. Prob (toxicity) = pdose ^ { exp(alpha) }, where pdose is the prior probability of DLT at the dose. Alpha is the parameter for the dose-toxicity curve. The prior distribution for alpha is assumed to be normal with mean 0 and standard deviation of 1.34.

10.4. Target toxicity level (TTL) and Maximum Tolerated Dose (MTD)

In this trial, only minimum toxicity is acceptable. The TTL is set to be at 10%. The MTD is defined as the highest dose level tested in which 10% of the patients develop DLT.

10.5. Dose Spacing

DOTAP:cholesterol-fus 1 toxicity during cycle 1 will be used to determine the next dose level. Toxicities or adverse events that are judged by the PI to be more likely due to the patient's underlying medical conditions or to other concurrent medications rather than to DOTAP:cholesterol-fus1 will not be used to guide decisions re subsequent dose escalations in future patients. Beginning with the starting dose, the subsequent dose level will be doubled if we observe no study-drug-related toxicities in all patients treated at that level. If we see grade 1 or 2 toxicities that are judged by the PI to be possibly, probably or definitely study-drug-related (and not judged by the PI to be more likely due to underlying conditions or concurrent medications), the next dose increment will be set at 50%. If we observe any DLT, all the subsequent dose increments will be set at 25%. Note that the above variable dose increment scheme sets the next dosage to be used should the observed result based on the current data lead to dose escalation recommended by CRM. It does not mean that when a DLT is observed, the next patient cohort will be treated at a 25% higher dose. On the contrary, the CRM will most likely recommend dose reduction when any DLT is observed. The specification of 25% dose increase upon observing any DLT is for the completeness of the dose specification. Because the TTL is set to be at 10%, dose escalation could occur if we observe only 1 DLT in 12 or more patients.

In addition, dose increase by 25% in a subsequent cohort could happen after observing a single patient with DLT when there was a previous dose reduction due to an observed single DLT with at least 6 patients accrued to the reduced dose level without observation of DLT, and after at least 15 patients are accrued to the dose level in which a single DLT was observed (before dose reduction) and the remaining 14 patients enrolled at the dose level do not experience DLT.

Without prior data in humans (similar to many of the other first human trials in drug development), we consider the variable dose increment to be a reasonable and efficient scheme for choosing the dose. With 7 dose levels, the maximum dosage could be as high as 1.28 mg/Kg. The preclinical data in monkeys and mice differ to some extent, making it difficult to predict prior probability of DLT. In monkeys, there was 1 death (1 out of 2 animals) at the highest dose level that was tested, 0.6 mg/Kg, and there were no deaths (0 out of 6 animals) at the lower dose level, 0.2 mg/Kg. Based on these monkey toxicology data, it seems unlikely that we will reach the maximum dosage level (1.28 mg/Kg). In mice there were no deaths (0 out of 15) at 0.5 mg/Kg and 1 death (1 out of 15) at 2 mg/Kg. Should it happen that MTD is not reached in the 7th dose, consideration will be given to amending the trial to study higher doses. New prior toxicities will be calculated based on accumulated data by that time.

10.6. Dose Escalation Scheme

Prior to proceeding to the next dose level and treating patients at this dose level, the Principal Investigator will submit a completed cohort summary that contains the PI analysis, to the sponsor (MDACC).

(a) The first patient will be treated at the lowest dose level.
(b) The cohort size is 3, i.e., a cohort of 3 patients will be treated at each dose level.
(c) After treating 3 patients at a given dose level, the patients will be observed for 2 weeks to evaluate the toxicity. The information of whether the patients develop DLT will be recorded for computing the posterior probability of toxicity given the prior and the data. The next cohort of patients will be treated at the dose level having the posterior probability of DLT closest to the pre-specified TTL.
(d) All the patients will be treated in a dose-escalation fashion starting from the lowest level. The next dose level can be moved up if indicated by the calculation of the posterior distribution but no skipping of doses is allowed.
(e) A "Look-ahead" option will be used. Specifically, instead of filling the cohort with 3 patients, dose reduction will be immediately applied if the first patient treated in a new dose develops DLT and CRM recommends dose reduction even if treating two more patients at the same dose produces no DLT.
(f) No intra-patient dose escalation is allowed.
(g) In the event of two instances of acute or delayed (observed beyond the initial 2 weeks) drug related (probable or definite) dose-limiting toxicities accrual will be halted and the FDA consulted.

10.7. Stopping Rule

A maximum of 51 patients will be treated in this Phase I trial. Under the dose escalation scheme defined above, patients will be treated at a dose level closest to the current estimate of the MTD. No early stopping is defined. Once the MTD is reached an additional 6 patients will be treated at that dose level.

10.8. Operating Characteristics

The prior probability of DLT at each dose level is 0.01, 0.02, 0.04, 0.07, 0.10, 0.15, and 0.25, respectively. The selection of prior probability is the best projection based on our experience with the regimen. In order to assure the success of the trial under different situations, three scenarios (scenario 1: expected result, scenario 2: regimen is less toxic than scenario 1, scenario 3: regimen is more toxic than scenario 1) are evaluated in simulation studies. CRM produces reasonable operating characteristics under all three scenarios. The probability of each dose level chosen as MTD (Column 4) under three scenarios is listed below.

Scenario 1:

Dose Level	Prior P(tox.)	True P(tox.)	Prob (MTD)	Average N
1	0.01	0.01	0.00	3.36
2	0.02	0.02	0.07	4.59
3	0.04	0.04	0.17	5.73
4	0.07	0.07	0.25	6.39
5	0.10	0.10	0.27	4.95
6	0.15	0.15	0.15	3.33
7	0.25	0.25	0.09	1.65

Scenario 2:

Dose Level	Prior P(tox.)	True P(tox.)	Prob (MTD)	Average N
1	0.01	0.01	0.00	3.36
2	0.02	0.02	0.06	4.35
3	0.04	0.03	0.09	4.41
4	0.07	0.05	0.11	5.61
5	0.10	0.07	0.34	5.13
6	0.15	0.10	0.21	4.20
7	0.25	0.20	0.19	2.94

Scenario 3:

Dose Level	Prior P(tox.)	True P(tox.)	Prob (MTD)	Average N
1	0.01	0.01	0.01	3.96
2	0.02	0.05	0.29	8.04
3	0.04	0.10	0.37	8.58
4	0.07	0.15	0.31	6.78
5	0.10	0.30	0.02	2.04
6	0.15	0.50	0.00	0.60
7	0.25	0.70	0.00	0.00

Due to the low TTL of 10% chosen, the CRM does not choose the exact dose level with high probability. However, the probability of selecting doses with TTL between 5% and 15% is reasonably high. The probability of choosing a highly toxic level (P(tox) >= 0.30) as MTD is very low (<=2%).

We use the computer program crm.exe, which can be downloaded from:
http://odin.mdacc.tmc.edu/anonftp/
The description is given below.
Continual Reassessment Method (CRM) For Dose-finding In Phase I Clinical Trials:
crm is version 1.0 of a menu-driven computer program which implements the Continual Reassessment Method (CRM) for dose-finding in Phase I clinical trials. It is designed for use by physicians, research nurses and statisticians. It provides a means to log in patients as they are accrued to the trial, to record their outcomes from treatment ("toxicity"' or "no toxicity"). The program selects doses for successive cohorts of patients based on the current data using the Bayesian method underlying the CRM.

REFERENCE

O'Quigley, J., Pepe, M. Fisher, L. (1990) Continual reassessment method: a practical design for phase I clinical trials in cancer. Biometrics 46, 33-48.

11.0. **Ethical and Regularity Considerations**

The final approved protocol and the informed consent statement to be used in the study will be reviewed by a properly constituted IRB. The Board's decision concerning the conduct of the study will be made in writing to the investigator and a copy of this decision will be provided to MDACC.

Particular attention is drawn to the FDA's regulation regarding IRBs. By signing the Statement of Investigator form (Form 1572), the investigator provides MDACC the necessary assurance that an IRB is responsible for the initial and continuing review and approval of the proposed clinical study in accordance with these regulations.

The investigator will agree to make required progress reports to the IRB, as well as report any serious adverse events, life-threatening problems, or deaths according to 21 CFR 312.64. The investigator will also inform the IRB of reports of serious adverse events (provided to the investigator by Sponsor) in other clinical studies conducted with the study medication.

11.1. Modification of the Protocol

Any changes to the protocol affecting study objectives, study design, patient population, study procedures, or significant administrative aspects will require a formal amendment to the protocol. Such amendments will be by the IRB prior to use in the study.

Administrative changes of the protocol are minor corrections and/or clarifications that have no effect on the way the study is to be conducted. These administrative changes will be agreed upon by the investigator and will be documented in a memorandum.

11.2. Protocol Deviations

The investigator will not deviate from the protocol without prior written permission from the Sponsor and the IRB, except in medical emergencies. In the event of a medical emergency the IRB will be notified within three (3) business days of the protocol deviation.

11.3. Concomitant Medications

11.3.1 Allowed

Supportive treatment will be given as medically indicated. All concomitant medications will be specified in the case report form.

11.3.2 Not Allowed

Any treatment that might interfere with the biological activity, the efficacy, or the evaluation of the proposed study treatment is not allowed throughout patient participation in the study. Agents that may not be administered to patients while they are on this trial include other anticancer therapy and other investigational therapy. Patients should not receive corticosteroids except as follows: dexamethasone before and for 36 hours after DOTAP:cholesterol-fus 1 as outlined in section 5.1, physiologic maintenance doses of corticosteroids (where indicated because of hypoadrenalism), inhaled steroids for as indicated for treatment of asthma or COPD, and short courses of corticosteroids as clinically indicated to treat relevant acute medical events.

11.4. Biosafety Procedures

A copy of the biosafety procedures approved by the IRB and/or Safety Committee will be kept on file in the Investigator Study File and the Pharmacy Study File. This document will be updated and revised as necessary to remain current with institutional guidelines.

11.5. Pregnancy

The risk to a fetus is unknown, and information from animal studies may or may not predict what will happen in humans. Patients in this study should make every effort to avoid pregnancy both for themselves and with their partner while on the study. If pregnancy occurs in a subject or their partner, the investigator must notify the Principal Investigator immediately.

11.6. Case Report Forms

All data will be entered into PDMS, which will serve as the electronic case report form (CRF).

11.7. Monitoring, Auditing, and Inspecting

The investigator will permit monitoring of the study as frequently as is deemed necessary to determine that data recording and protocol adherence are satisfactory.

During site visits, the study monitor will review original patient records, drug accountability records, and document retention (Investigator Study File). The investigator should allow adequate time for these visits. The investigator should also ensure that the monitor is given access to source documents (i.e., hospital or private charts, laboratory records, radiographic films, photographs, appointment books, etc.) of the patient which support data entered in the case report forms. The investigator and study staff will be expected to cooperate with the monitor and to provide all missing information wherever possible.

Participation in this study implies acceptance of potential inspection by FDA authorities.

11.8. Termination of the Study

If, in the judgment of the investigators, the continued exposure to the study vector represents a significant risk to patients, the study will be terminated.

11.9. Records Retention

It is the investigator's responsibility that sufficient information pertaining to the identity of the patients will be retained, so that any FDA representative may access this information, should the need arise. Copies of all pertinent information, including patient name, patient identification number, and individual patient data records, will be retained in a confidential manner by the investigator for a minimum period of two years following the date of marketing application approval for the drug for the indication for which it was being investigated. If no application is filed, or if the application is not approved for the indication, the records will be kept for two years after the investigation is discontinued and the FDA is notified of that fact.

11.10. Use of Information and Publication

Neither the complete nor any part of the results of the study carried out under this protocol nor any of the information provided by the Sponsor to the investigator for the purposes of performing the study will be published by or passed to any third party without prior written consent of the Sponsor.

The investigator is obliged to provide the Sponsor with complete test results and all data derived from the study. Only the Sponsor may make information obtained during the study available to physicians or regulatory agencies, except as required by regulation.

12.0 References

1. Wingo PA, Tang T, Bolden S: Cancer statistics, 1995. CA Cancer J Clin 1995;45:8-30.

2. Bishop JM: Molecular themes in oncogenesis. Cell 1991;64:235-248.

3. Minna J: Summary of the role of dominant and recessive oncogenes in the pathogenesis of lung cancer and the application of this knowledge in translational research. In: Pass H, Mitchell J, Johsnon D, Turrisi A, eds. Philadelphia: JB Lippincott Co., 1994:

4. Gazdar AF, Bader S, Hung J, et al: Molecular genetic changes found in human lung cancer and its precursor lesions. In: AnonymousMolecular genetic changes found in human lung cancer and its precursor lesions. 1995:565-572.

5. Whang-Peng J, Kao-Shan CS, Lee EC, et al: Specific chromosome defect associated with human small-cell lung cancer; deletion 3p(14-23). Science 1982;215:181-182.

6. Naylor SL, Johnson BE, Minna JD, Sakaguchi AY: Loss of heterozygosity of chromosome 3p markers in small-cell lung cancer. Nature 1987;329:451-454.

7. Brauch H, Tory K, Kotler F, et al: Molecular mapping of deletion sites in the short arm of chromosome 3 in human lung cancer. Genes, Chromosomes & Cancer 1990;1:240-246.

8. Whang-Peng J, Knutsen T, Gazdar A, et al: Nonrandom structural and numerical chromosome changes in non-small-cell lung cancer. Genes Chromosomes Cancer 1991;3:168-188.

9. Daly MC, Xiang RH, Buchhagen D, et al: A homozygous deletion on chromosome 3 in a small cell lung cancer cell line correlates with a region of tumor suppressor activity. Oncogene 1993;8:1721-1729.

10. Rabbitts P, Douglas J, Daly M, et al: Frequency and extent of allelic loss in the short arm of chromosome 3 in nonsmall-cell lung cancer. Genes Chromosomes Cancer 1989;1:95-105.

11. Kok K, Osinga J, Carritt B, et al: Deletion of a DNA sequence at the chromosomal region 3p21 in all major types of lung cancer. Nature 1987;330:578-581.

12. Hibi K, Takahashi T, Yamakawa K, et al: Three distinct regions involved in 3p deletion in human lung cancer. Oncogene 1992;7:445-449.

13. Yokota J, Wada M, Shimosato Y, Terada M, Sugimura T: Loss of heterozygosity on chromosomes 3, 13, and 17 in small- cell carcinoma and on chromosome 3 in adenocarcinoma of the lung. Proc Natl Acad Sci U S A 1987;84:9252-9256.

14. Horio Y, Takahashi T, Kuroishi T, et al: Prognostic significance of p53 mutations and 3p deletions in primary resected non-small cell lung cancer. Cancer Res 1993;53:1-4.

15. Devilee P, Van den Broek M, Kuipers-Dijkshoorn N, et al: At least four different chromosomal regions are involved in loss of heterozygosity in human breast carcinoma. Genomics 1989;5:554-560.

16. Jones MH, Koi S, Fujimoto I, Hasumi K, Kato K, Nakamura Y: Allelotype of uterine cancer by analysis of RFLP and microsatellite polymorphisms: frequent loss of heterozygosity on chromosome arms 3p, 9q, 10q, and 17p. Genes, Chromosomes & Cancer 1994;9:119-123.

17. Andersen TI, Gaustad A, Ottestad L, et al: Genetic alterations of the tumour suppressor gene regions 3p, 11p, 13q, 17p, and 17q in human breast carcinomas. Genes, Chromosomes & Cancer 1992;4:113-121.

18. Brooks JD, Bova GS, Marshall FF, Isaacs WB: Tumor suppressor gene allelic loss in human renal cancers. Journal of Urology 1993;150:1278-1283.

19. Kohno T, Takayama H, Hamaguchi M, et al: Deletion mapping of chromosome 3p in human uterine cervical cancer. Oncogene 1993;8:1825-1832.

20. Li X, Lee NK, Ye YW, et al: Allelic loss at chromosomes 3p, 8p, 13q, and 17p associated with poor prognosis in head and neck cancer. J Natl Cancer Insti 1994;86:1524-1529.

21. Maestro R, Gasparotto D, Vukosavljevic T, Barzan L, Sulfaro S, Boiocchi M: Three discrete regions of deletion at 3p in head and neck cancers. Cancer Res 1993;53:5775-5779.

22. Thrash-Bingham CA, Greenberg RE, Howard S, et al: Comprehensive allelotyping of human renal cell carcinomas using microsatellite DNA probes. Proc Natl Acad Sci U S A 1995;92:2854-2858.

23. Wu CL, Sloan P, Read AP, Harris R, Thakker N: Deletion mapping on the short arm of chromosome 3 in squamous cell carcinoma of the oral cavity. Cancer Res 1994;54:6484-6488.

24. Zeiger MA, Gnarra JR, Zbar B, Linehan WM, Pass HI: Loss of heterozygosity on the short arm of chromosome 3 in mesothelioma cell lines and solid tumors. Genes, Chromosomes & Cancer 1994;11:15-20.

25. Sundaresan V, Ganly P, Hasleton P, et al: p53 and chromosome-3 abnormalities, characteristic of malignant lung tumours, are detectable in preinvasive lesions of the bronchus. Oncogene 1992;7:1989-1997.

26. Hung J, Kishimoto Y, Sugio K, et al: Allele-specific chromosome 3p deletions occur at an early stage in the pathogenesis of lung carcinoma. JAMA 1995;273:558-563.

27. Sozzi G, Miozzo M, Pastorino U, et al: Genetic evidence for an independent origin of multiple preneoplastic and neoplastic lung lesions. Cancer Res 1995;55:135-140.

28. Sanchez Y, Killary A: Definition of a tumor suppressor gene on human chromosome 3 involved in renal cell carcinoma. Am J Hum Genet 1992;51:A70

29. Killary AM, Wolf ME, Giambernardi TA, Naylor SL: Definition of a tumor suppressor locus within human chromosome 3p21-p22. Proc Natl Acad Sci U S A 1992;89:10877-10881.

30. Imreh S, Kholodnyuk I, Allikmetts R, Stanbridge EJ, Zabarovsky ER, Klein G: Nonrandom loss of human chromosome 3 fragments from mouse-human microcell hybrids following progressive growth in SCID mice. Genes, Chromosomes & Cancer 1994;11:237-245.

31. Rimessi P, Gualandi F, Morelli C, et al: Transfer of human chromosome 3 to an ovarian carcinoma cell line identifies three regions on 3p involved in ovarian cancer. Oncogene 1994;9:3467-3474.

32. Satoh H, Lamb PW, Dong JT, et al: Suppression of tumorigenicity of A549 lung adenocarcinoma cells by human chromosomes 3 and 11 introduced via microcell-mediated chromosome transfer. Molecular Carcinogenesis 1993;7:157-164.

33. Yoshida MA, Shimizu M, Ikeuchi T, Tonomura A, Yokota J, Oshimura M: In vitro growth suppression and morphological change in a human renal cell carcinoma cell line by the introduction of normal chromosome 3 via microcell fusion. Molecular Carcinogenesis 1994;9:114-121.

34. Swisher SG, Roth JA, Nemunaitis J, et al: Adenoviral-mediated p53 gene transfer in advanced non-small cell lung cancer. J Natl Cancer Inst 1999;91:763-771.

35. Clayman GL, El-Naggar AK, Lippman SM, et al: Adenovirus-mediated p53 gene transfer in patients with advanced recurrent head and neck squamous cell carcinoma. J Clin Oncol 1998;16:2221-2232.

36. Yang JP, Huang L: Overcoming the inhibitory effect of serum on lipofection by increasing the charge ratio of cationic liposome to DNA. Gene Ther 1997;4:950-960.

37. Felgner PL, Gadek TR, Holm M, et al: Lipofection: a highly efficient, lipid-mediated DNA-transfection procedure. Proc Natl Acad Sci U S A 1987;84:7413-7417.

38. Zhu N, Liggitt D, Liu Y, Debs R: Systemic gene expression after intravenous DNA delivery into adult mice. Science 1993;261:209-211.

39. Philip R, Liggitt D, Philip M, Dazin P, Debs R: In vivo gene delivery. Efficient transfection of T lymphocytes in adult mice. J Biol Chem 1993;268:16087-16090.

40. Solodin I, Brown CS, Bruno MS, et al: A novel series of amphiphilic imidazolinium compounds for in vitro and in vivo gene delivery. Biochemistry 1995;34:13537-13544.

41. Liu Y, Liggitt D, Zhong W, Tu G, Gaensler K, Debs R: Cationic liposome-mediated intravenous gene delivery. J Biol Chem 1995;270:24864-24870.

42. Shao L, Gao Z, Yang N, Wei G, Wang Y, Cheng C: Results of surgical treatment in 6,123 cases of carcinoma of the esophagus and gastric cardia. J Surg Oncol 1989;42:170-174.

43. Thierry AR, Lunardiiskandar Y, Bryant JL, Rabinovich P, Gallo RC, Mahan LC: Systemic gene therapy: Biodistribution and long-term expression of a transgene in mice. Proc Natl Acad Sci U S A 1995;92:9742-9746.

44. Tsukamoto M, Ochiya T, Yoshida S, Sugimura T, Terada M: Gene transfer and expression in progeny after intravenous DNA injection into pregnant mice. Nature Genetics 1995;9:243-248.

45. Aksentijevich I, Pastan I, Lunardi-Iskandar Y, Gallo RC, Gottesman MM, Thierry AR: In vitro and in vivo liposome-mediated gene transfer leads to human MDR1 expression in mouse bone marrow progenitor cells. Human Gene Ther 1996;7:1111-1122.

46. Dow S, Fradkin L, Liggitt D, Willson A, Heath T, Potter T: Lipid-DNA complexes induce potent activation of innate immune responses and antitumor activity when administered intravenously. The Journal of Immunology 1999.
