# Supplementary material for: Phase I Clinical Trial of Systemically Administered TUSC2(FUS1)-Nanoparticles Mediating Functional Gene Transfer in Humans
Source: PLoS One. 2012 Apr 25;7(4):e34833. doi: 10.1371/journal.pone.0034833 (PMC3338819; doi:10.1371/journal.pone.0034833)
Supplement: Figure S2 — Positions and direction of DNA sequencing primers for pLJ143/KGB2/FUS1 vector. The entire DNA sequence of the plasmid vector is determined by automated DNA sequencing using the DNA Sequencing Core Facility at the M.D. Anderson Cancer Center. The positions and directions of the primers that are used for DNA sequencing are shown. The complete DNA sequence of pLJ143/KGB2/FUS1 plasmid vector are shown in Table 2. (DOCX) [file pone.0034833.s004.docx]

**Figure S2**.

***Sequencing primers for pLJ143***

A41: **5’ CGAGTCTCCACGTAAACGGT 3’**

A42**: 5’ ATGCCTGCTATTGTCTTCCC 3’**

A43 **5’ CCTTGAGCCTGGCGAACAGT 3’**

A44: **5’ AGCACCGCCTACATACCTCG 3’**

A45:  **5’ CATGTACTGGGCATAATGCC 3’**

A46**: 5’ GGAGGGGCAAATCCAGGCTT 3’**

S42**: 5’ CTAGTTGCCAGCCATCTGTT 3’**

S43**: 5’ CGGCTGCATACGCTTGATCC 3’**

S44: **5’ AAGCTGGGCTGTGTGCACGA 3’**

S45:  **5’ GGGGACTTTGACCGTTTACG 3’**

S46**: 5’ GCTACAACCACTAGCACGGCTGACG 3’**
