# Supplementary figures and images for: Phase I Clinical Trial of Systemically Administered TUSC2(FUS1)-Nanoparticles Mediating Functional Gene Transfer in Humans
Source: PLoS One. 2012 Apr 25;7(4):e34833. doi: 10.1371/journal.pone.0034833 (PMC3338819; doi:10.1371/journal.pone.0034833)

**Figure S3A.**

**
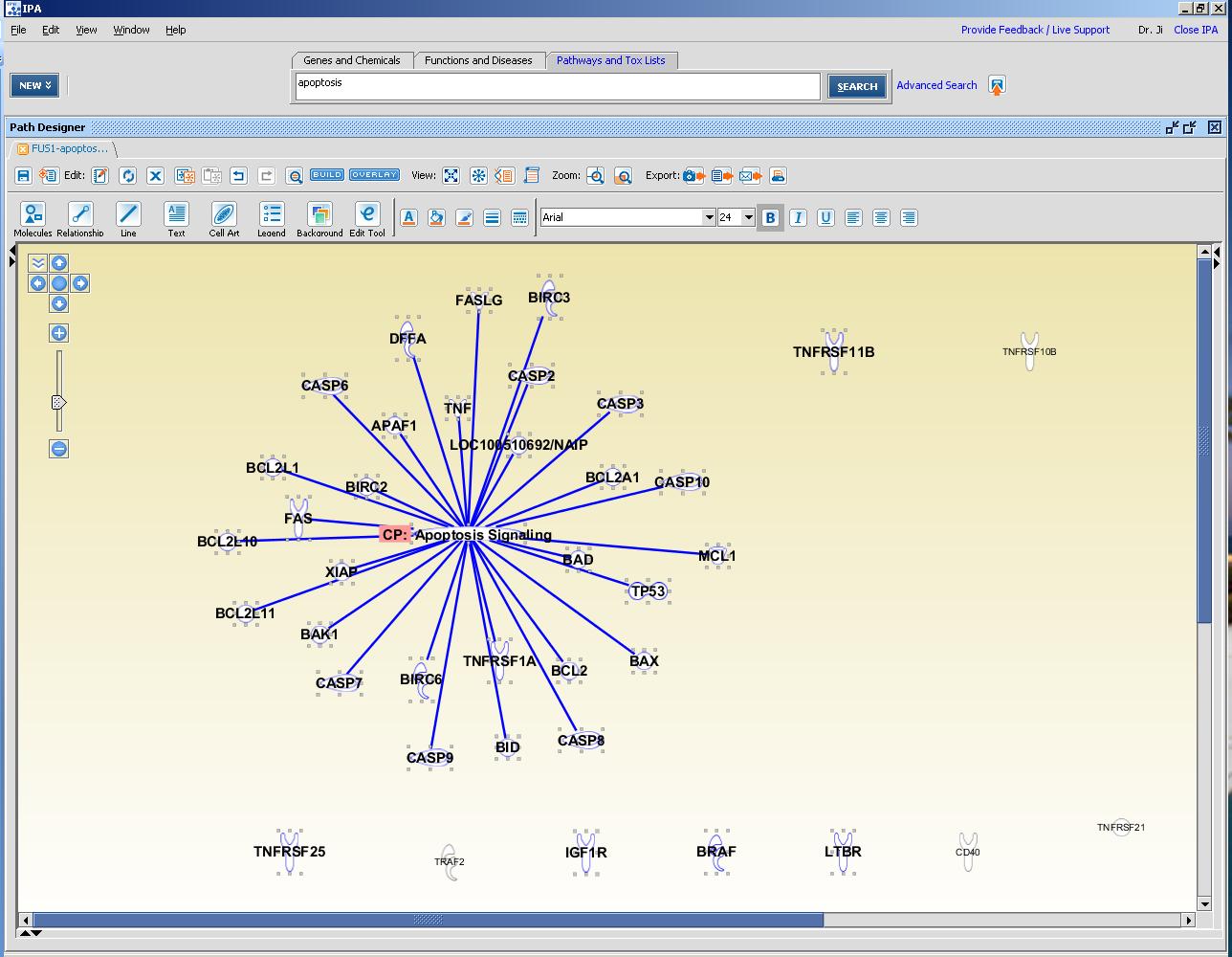
**

**Figure S3B.**


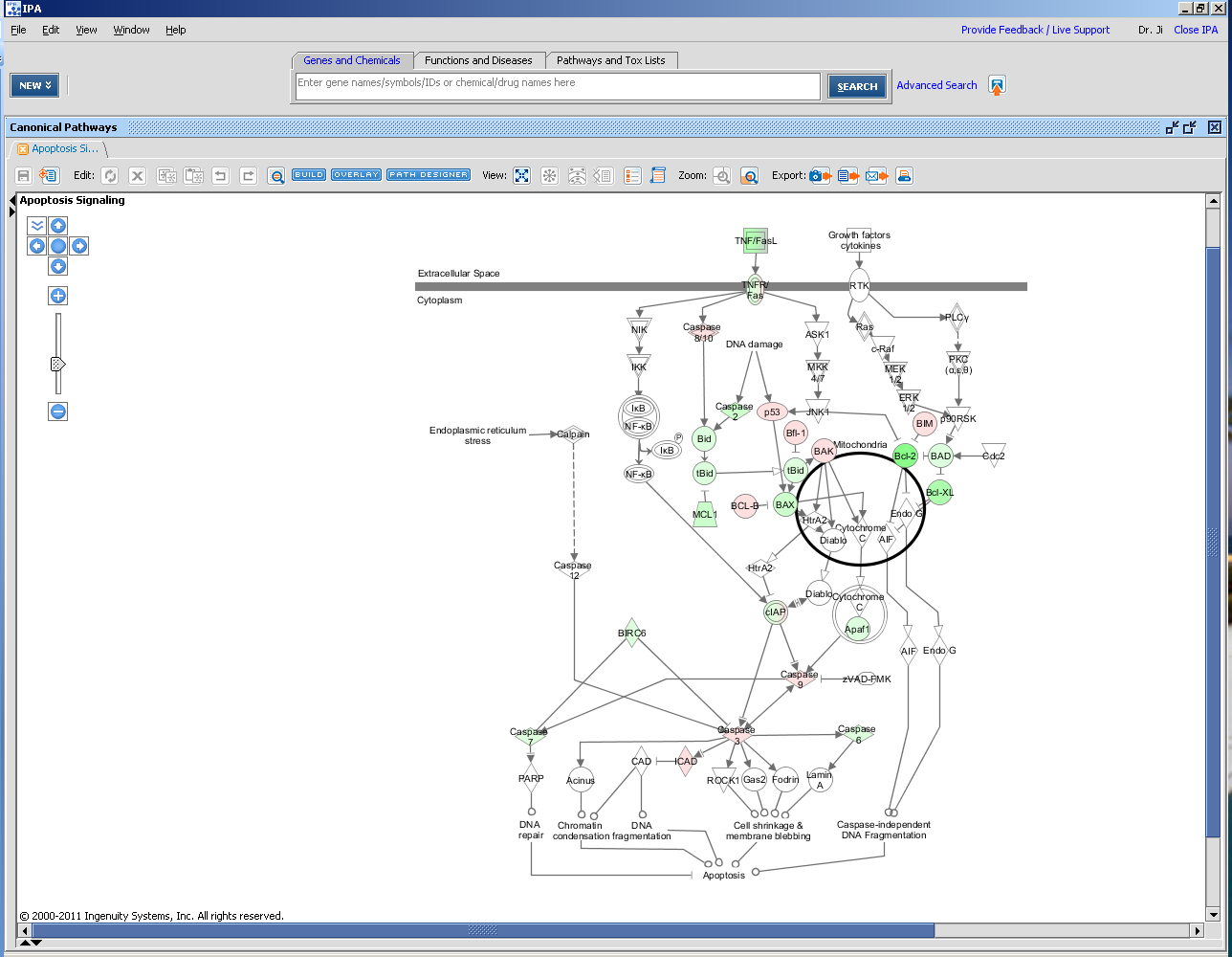


**Patient 13**


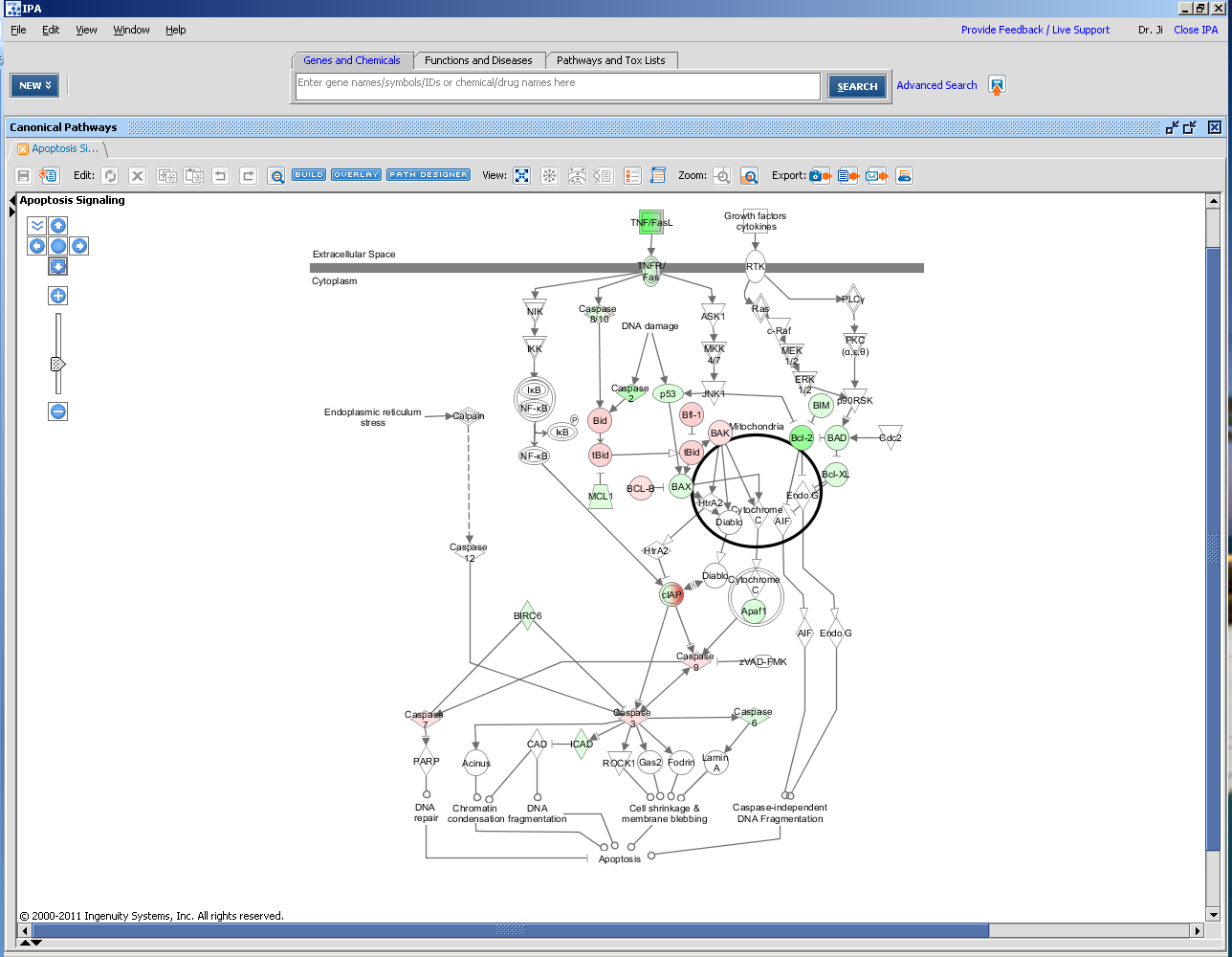


**Patient 31**

Supplement: Figure S3 — Genes which show significant changes in expression for both patients 13 and 31 by IPA biomarker comparison analysis. A: Change in apoptosis pathway mRNAs analyzed in pre and post-treatment biopsy specimens from patients 13 and 31 using SA Apoptosis Signaling Nano-scale PCR Array. Criteria for selection are presented in the Ingenuity Apoptosis Pathway Analysis section of the Information S1. Increased exogenous TUSC2 mRNA expression was detected in the post-treatment biopsy from both patients. B: Canonical apoptosis pathway gene expression pertubations following TUSC2-nanoparticle treatment. Canonical apoptosis pathway gene expression pertubations following TUSC2-nanoparticle treatment as detected by SA PRC Array and IPA Analysis. Molecules are represented as nodes, and the biological relationship between two nodes is represented as an edge (Line). The intensity of the node color indicates the degree of up- (red) or down- (green) regulation Nodes are displayed using various shapes that represent the functional class of the gene products. Edges are displayed with various labels that describe the nature of the relationship between the nodes (e.g., P for phosphorylation, T for transcription). The identified nodes indicate perturbation of elements of the intrinsic and extrinsic apoptotic pathways following treatment with DOTAP:chol-TUSC2. (DOCX) [file pone.0034833.s005.docx]
