# Supplementary material for: Phase I Clinical Trial of Systemically Administered TUSC2(FUS1)-Nanoparticles Mediating Functional Gene Transfer in Humans
Source: PLoS One. 2012 Apr 25;7(4):e34833. doi: 10.1371/journal.pone.0034833 (PMC3338819; doi:10.1371/journal.pone.0034833)
Supplement: Table S1 — Dose-Escalation Scheme. (DOCX) [file pone.0034833.s006.docx]

**Table S1:**  **Dose-Escalation Scheme**
